# Cholinergic modulation of hippocampal network function

**DOI:** 10.3389/fnsyn.2013.00002

**Published:** 2013-07-30

**Authors:** Leonor M. Teles-Grilo Ruivo, Jack R. Mellor

**Affiliations:** Centre for Synaptic Plasticity, School of Physiology and Pharmacology, University of Bristol, University WalkBristol, UK

**Keywords:** synaptic plasticity, acetylcholine, medial septum, hippocampus, septohippocampal pathway, memory

## Abstract

Cholinergic septohippocampal projections from the medial septal area to the hippocampus are proposed to have important roles in cognition by modulating properties of the hippocampal network. However, the precise spatial and temporal profile of acetylcholine release in the hippocampus remains unclear making it difficult to define specific roles for cholinergic transmission in hippocampal dependent behaviors. This is partly due to a lack of tools enabling specific intervention in, and recording of, cholinergic transmission. Here, we review the organization of septohippocampal cholinergic projections and hippocampal acetylcholine receptors as well as the role of cholinergic transmission in modulating cellular excitability, synaptic plasticity, and rhythmic network oscillations. We point to a number of open questions that remain unanswered and discuss the potential for recently developed techniques to provide a radical reappraisal of the function of cholinergic inputs to the hippocampus.

## Introduction

Neuromodulation is a feature of complex nervous systems that is proposed to play an essential role in behavior allowing anatomically defined neural circuitry to be re-purposed, thereby enabling rapid adaptation in response to external stimuli (Katz, 2011; Marder, 2012). Neuromodulation is achieved through the orchestrated activity of an ensemble of central modulators; acetylcholine, serotonin, dopamine, norepinephrine, and numerous neuropeptides are released in response to specific stimuli and have long-lasting, and often long-range, diffuse effects on central processing.

The neuromodulator acetylcholine has been extensively studied due to its prominent role in attention, learning, and synaptic plasticity (Everitt and Robbins, 1997; Hasselmo, 2006; Micheau and Marighetto, 2011). Acetylcholine is also implicated in the etiology of neurological disorders such as Alzheimer's disease (Bartus et al., 1982; Raedler et al., 2007; Schliebs and Arendt, 2011), which has led to targeting of the cholinergic system for the development of cognitive enhancers such as acetylcholinesterase inhibitors to combat dementia. Given the central role the hippocampus plays in declarative memory formation and the strong cholinergic input to the hippocampus from the septohippocampal pathway, it is tempting to hypothesize that this input is critical for memory processes (Dutar et al., 1995; Hasselmo, 2006; Drever et al., 2011). However, evidence to support this hypothesis is inconclusive. For example, functional studies have provided conflicting information on the effects of damage to the septohippocampal cholinergic system (Kelsey and Landry, 1988; Lee et al., 1994; Dutar et al., 1995; McMahan et al., 1997; McGaughy et al., 2000; Lecourtier et al., 2011). Not all studies show lesions to the septohippocampal pathway, and the consequent loss of cholinergic neurons, to be associated with deficits in memory functions usually associated with aging-related disabilities or neurodegenerative diseases (Fibiger, 1991; Muir, 1997; Davis et al., 1999; McGaughy et al., 2000; Micheau and Marighetto, 2011; Schliebs and Arendt, 2011) Similarly, pharmacological or genetic inhibition of acetylcholine receptors cause memory deficits but it is often unclear which receptor subtypes are involved and which part of the brain they are located in De Rosa and Hasselmo (2000); Anagnostaras et al. (2003); Warburton et al. (2003); Atri et al. (2004); Wess (2004). The recent generation of conditional knockout mice may resolve some of these issues (Wess, 2012).

Septohippocampal cholinergic fibers ramify extensively throughout the hippocampus with release sites often occurring without identified apposite postsynaptic entities. This supports the concept of a diffuse projection engaged in long-lasting effects (Vizi and Kiss, 1998; Zoli et al., 1999). However, high resolution information on the spatial and temporal profile of acetylcholine release in the hippocampus during awake behavior is not currently available making it hard to define specific functions of acetylcholine release. Recent high resolution measurements of acetylcholine release in the cortex have demonstrated that release may be precisely timed (Parikh et al., 2007; Howe et al., 2013), leading to a reappraisal of the role of acetylcholine in network function.

Synaptic plasticity is often considered the cellular and molecular correlate of learning and memory. In this context, electrophysiological data for the role of acetylcholine in hippocampal synaptic plasticity is also mixed. Under a variety of *in vitro* and *in vivo* conditions, acetylcholine either facilitates or directly causes hippocampal long-term potentiation (LTP) or depression (LTD) (Markram and Segal, 1990c; Leung et al., 2003; Ovsepian et al., 2004; Shinoe et al., 2005; Isaac et al., 2009; Buchanan et al., 2010; Jo et al., 2010; Gu and Yakel, 2011; Sugisaki et al., 2011), implicating a role for cholinergic input in synaptic plasticity but leaving open the questions of exactly how and by what mechanisms.

In addition to a role in synaptic plasticity, it has been proposed that cholinergic septohippocampal projections are critical for generating (Buzsaki et al., 1983; Bland and Colom, 1993) and phasing hippocampal theta and gamma oscillatory activity (Stewart and Fox, 1990; Lee et al., 1994; Bland et al., 1999; Buzsaki, 2002), therefore playing a pivotal role in processes associated with learning and memory consolidation (Buzsaki, 2005; Lecourtier et al., 2011). Although there are strong correlations between behavioral state, rhythmic network oscillations and cholinergic input to the hippocampus (Zhang et al., 2010, 2011), the mechanisms underlying these processes remain unclear.

The intimate relationship between neuronal activity, brain oscillations and cholinergic neuromodulation has probably been a hindering factor to the dissection of roles played by the septohippocampal cholinergic system in modulating theta and gamma oscillations, synaptic plasticity, and memory formation in the hippocampus. To make a detailed analysis of these roles we require tools that allow specific interventions and measurement of cholinergic function.

## Anatomical organization of the septohippocampal pathway

**Figure 1:**
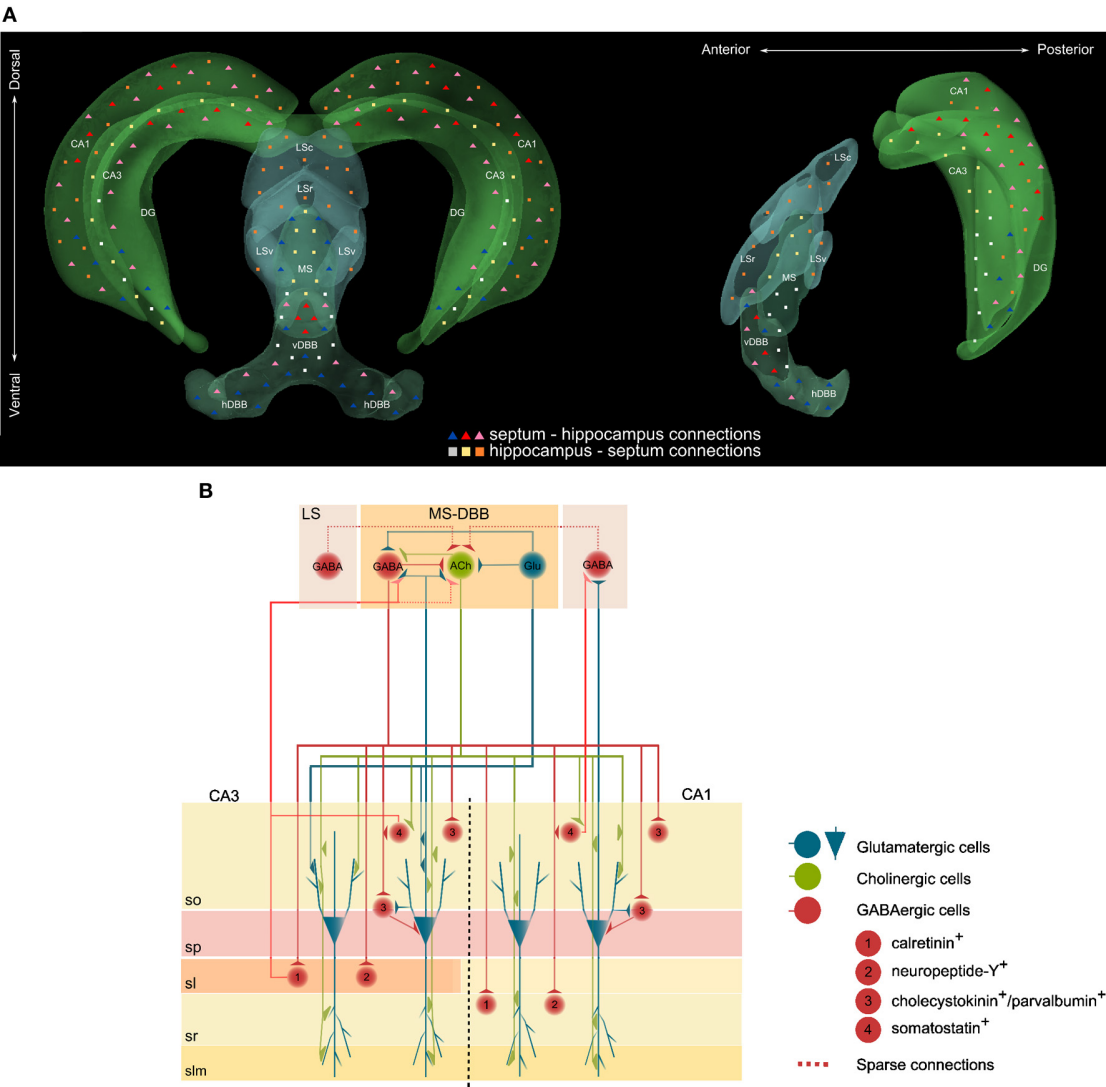
**(A)** Schematic representation of the reciprocal connections between the septum and the hippocampus. In the coronal (left) and saggital (right) views, triangles represent connections from the septum to the hippocampus and squares represent connections from the hippocampus to the septum. Color-code specifies sub-regions where cell bodies are located and where axonal projections terminate. Abbreviations: LSc, lateral septum caudal; LSr, lateral septum rostral; LSv, lateral septum ventral; MS, medial septum; vDBB, vertical band of the diagonal band of Broca; hDBB, horizontal band of the diagonal band of Broca. **(B)** Connections between glutamatergic, cholinergic and GABAergic neurons in the septohippocampal pathway. Abbreviations: Glu, glutamate; ACh, acetylcholine; LS, lateral septum; MS-DBB, medial septum-diagonal band of Broca; so, stratum oriens; sp, stratum pyramidale; sl, stratum lucidum; sr, stratum radiatum; slm, stratum lacunosum-moleculare.

The function of neuromodulatory systems is largely defined by the anatomy of their projections. The septohippocampal pathway is the main source of cholinergic innervation to the hippocampus (Lewis and Shute, 1967; Dutar et al., 1995) and has been anatomically mapped with its afferent and efferent projections, and respective cellular targets, characterized in detail. The principal divisions of the septal area include the medial and lateral septal nuclei and the nucleus of the diagonal band of Broca (DBB), which is further subdivided into vertical and horizontal limbs. Via the fimbria and dorsal fornix, the hippocampus is reciprocally connected to the medial septum forming a single continuous anatomical structure with functionally coupled components. Studies combining retrograde tracing, lesions, and immunocytochemistry demonstrated that the septohippocampal projection is topographically organized along the mediolateral and rostrocaudal axes—laterally located nuclei project ventrally whereas rostral neurons extend their axons rostrally into the hippocampus (Meibach and Siegel, 1977; Sakanaka et al., 1980; Amaral and Kurz, 1985) (Figure 1). Within the hippocampal formation, the CA fields and the dentate gyrus are innervated by septal fibers in a laminar pattern. The CA1 pyramidal and dentate granule cell layers in the dorsal hippocampus receive inputs from neurons located along the midline of the vDBB; cells located immediately lateral to the midline of the DBB project through the medial part of the fimbria to all CA fields of the caudal region of the hippocampus; finally, cells in the ventral hippocampal formation are supplied by both the DBB and the intermediolateral septum (McKinney et al., 1983; Nyakas et al., 1987) (Figure 1).

The degree of target cell specificity observed in septohippocampal neurons is dependent on their neurotransmitter content. Septohippocampal projections encompass immunohistochemically distinct cholinergic, GABAergic and glutamatergic neurons (Freund and Antal, 1988; Pepeu and Blandina, 1998; Manns et al., 2001; Gritti et al., 2006; Lecourtier et al., 2011). In addition, co-synthesis of glutamate in cholinergic and GABAergic neurons has also been reported (Manns et al., 2001; Gritti et al., 2006). Medial septal cholinergic terminals project to all regions of the hippocampus (Milner et al., 1983; Amaral and Kurz, 1985), targeting the stratum oriens of CA1 and CA3 subfields (Houser et al., 1983; Frotscher and Leranth, 1985; Matthews et al., 1987), where synaptic contacts are established with dendrites of pyramidal cells (Wainer et al., 1984), as well as cell bodies and dendrites of GABA- and somatostatin-containing interneurons (Frotscher and Leranth, 1985; Leranth and Frotscher, 1987; Yamano and Luiten, 1989; Cobb and Davies, 2005) and dentate granule cells (Nyakas et al., 1987). With a higher degree of target cell-type specificity, medial septal GABAergic fibers terminate on vasoactive intestinal polypeptide (VIP)-immunoreactive interneurons in strata pyramidale and lacunosum-moleculare of the CA1 (Papp et al., 1999) and on calretinin- and neuropeptide Y-immunoreactive GABAergic interneurons in the stratum radiatum of the CA1 and stratum lucidum of CA3 (Freund and Antal, 1988; Gulyas et al., 1990; Acsady et al., 1993; Takacs et al., 2008). Inhibitory inputs have also been shown to terminate on cholecystokinin-, somatostatin- and parvalbumin-containing neurons in the stratum oriens (Freund and Antal, 1988; Yamano and Luiten, 1989; Gulyas et al., 1990; Takacs et al., 2008). Medial septal glutamatergic neurons projecting to the hippocampus have recently been shown to provide functional excitatory inputs to CA3 pyramidal cells (Sotty et al., 2003; Manseau et al., 2005; Huh et al., 2010).

On the reciprocal side of the septohippocampal pathway, pyramidal and non-pyramidal cells from the CA1 project to the rostral and ventral parts of the lateral septum, whereas CA3 cells project to both the caudal part of the lateral septum and the MS-DBB in a topographical manner (Alonso and Kohler, 1982; Schwerdtfeger and Buhl, 1986; Leranth and Frotscher, 1989; Toth and Freund, 1992; Toth et al., 1993; Risold and Swanson, 1997; Gulyas et al., 2003; Thompson et al., 2008). The dorsal region of the CA3 innervates the dorsal and medial parts of the medial septum and the rostral and dorsal parts of the vDBB; conversely, axons of the ventral portion reach the lateral and ventral parts of the medial septum and the caudal and ventral parts of the vDBB (Gaykema et al., 1991).

Although to date our knowledge of intraseptal connectivity is limited, connections have been reported to include sparse GABAergic inputs from lateral to medial septal cholinergic neurons, reciprocal connections between medial septal cholinergic and GABAergic neurons and also glutamatergic neurons within the medial septum synapsing onto neighboring cholinergic and GABAergic neurons (Leranth et al., 1992; Hajszan et al., 2004; Manseau et al., 2005) (Figure 1).

This anatomical organization gives rise to the septohippocampal system, a long-range feedback loop between the hippocampus and medial septum. This feedback loop allows cholinergic, GABAergic and glutamatergic neuronal populations to interact and modulate rhythmic activity and synaptic plasticity in the hippocampus.

***Questions:*** *Although the anatomy of the septohippocampal cholinergic projection is well characterized, we know little about the functional output of these neurons. For example, can the septohippocampal cholinergic system be viewed as a single unit providing diffuse innervation of the hippocampus or are cholinergic neurons independent, targeting acetylcholine release to discrete cell types in specific areas of the hippocampus? What are the firing patterns of medial septal cholinergic neurons during different behavioral states? What are the functions of intraseptal connections? Is there interplay between intraseptal connections and afferent hippocampal inputs to regulate medial septal firing patterns?*

## Acetylcholine receptors

To understand the function of acetylcholine in the hippocampus it is necessary to know not only where it is released, but also the location and subtype of receptors it binds to and the effect of receptor activation on cellular and synaptic properties. Acetylcholine release within hippocampal circuits results in the activation of both muscarinic (mAChRs) and nicotinic (nAChRs) acetylcholine receptors, causing the subsequent modulation of cellular excitability and synaptic transmission. These two types of receptors are differentially expressed across the hippocampus (Table 1) and fulfill different functions.

**Table 1:** **Distribution of acetylcholine receptors in the hippocampus**.

**Receptor subtype**	**Hippocampal distribution**	**References**
M1	Soma, dendrites, and spines of excitatory neurons including granule neurons CA3 and CA1 pyramidal neurons.	Levey et al., 1995; Yamasaki et al., 2010; Cea-Del Rio et al., 2010, 2011; Dasari and Gulledge, 2011
	Neurons in the hilus.	
	Parvalbumin positive basket cells and cholecystokinin positive Schaffer collateral associated cells in CA1.	
M2	Presynaptic terminals of parvalbumin positive basket and axo-axonic cells in CA3 and CA1.	Levey et al., 1995; Hajos et al., 1998; Szabo et al., 2010
	Presynaptic terminals of septohippocampal cholinergic and non-cholinergic inputs.	
M3	Soma and dendrites of excitatory neurons including granule neurons CA3 and CA1 pyramidal neurons.	Levey et al., 1995; Cea-Del Rio et al., 2010, 2011; Dasari and Gulledge, 2011
	Cholecystokinin positive basket cells and Schaffer collateral associated cells in CA1.	
M4	Soma of non-pyramidal cells.	Levey et al., 1995; Dasari and Gulledge, 2011
	Presynaptic Schaffer collateral terminals.	
	Presynaptic localization on septal non-cholinergic inputs.	
M5	Limited protein identified.	Levey et al., 1991; Wall et al., 1994; Reever et al., 1997
α7	Presynaptic and postsynaptic at both glutamatergic and GABAergic synapses.	Ji and Dani, 2000; Alkondon and Albuquerque, 2001; Fabian-Fine et al., 2001; Sharma and Vijayaraghavan, 2003; Tang et al., 2011
	Postsynaptic at cholinergic synapses.	
α4β2	Soma of excitatory neurons.	
	Presynaptic on GABAergic terminals.	
α3β4	Presynaptic at glutamatergic and GABAergic terminals.	

Nicotinic acetylcholine receptors are ionotropic pentameric receptors made up of heteromeric or homomeric assemblies of α2−α10 and β2−β4 subunits. In the hippocampus, the nAChR subtypes predominantly expressed are α7, α4β2, and α3β4 (Dani and Bertrand, 2007; Albuquerque et al., 2009; Drever et al., 2011). α7 receptors are widely expressed in dentate granule cells, pyramidal cells and interneurons both pre- and postsynaptically (Fabian-Fine et al., 2001). However, fast α7-mediated synaptic currents have principally been shown to occur in interneurons and not in excitatory neurons (Frazier et al., 1998; Ji and Dani, 2000; Alkondon and Albuquerque, 2001; Kawai et al., 2002) although one study reports limited evidence in CA1 pyramidal neurons (Gu and Yakel, 2011). α4β2 receptors are expressed on interneuron axons terminating on excitatory and inhibitory neurons (Hill et al., 1993; Alkondon et al., 1999; Alkondon and Albuquerque, 2001; Graham et al., 2003; Bell et al., 2011). α3β4 receptors are expressed at inhibitory synapses contacting pyramidal neurons (Tang et al., 2011).

Activation of nAChRs results in direct Ca^2+^ influx through the channel pore and rapid membrane depolarization. The precise Ca^2+^ permeability of receptors depends on the subunit composition with α7 being the most permeable. Ca^2+^ accumulation in presynaptic terminals facilitates neurotransmitter release (Lena et al., 1993; McGehee et al., 1995; Wonnacott, 1997; Fu et al., 1998; Tang et al., 2011). Postsynaptically, cation flux through nAChRs mediates fast excitatory synaptic responses (Frazier et al., 1998; McQuiston and Madison, 1999; Ji and Dani, 2000; Alkondon and Albuquerque, 2001; Kawai et al., 2002; Wanaverbecq et al., 2007; Bell et al., 2011; Gu and Yakel, 2011; Tang et al., 2011). Fast membrane depolarization triggers activation of voltage-gated Ca^2+^ channels, second messenger systems involving cAMP (Margiotta et al., 1987; Sargent, 1993) and release from intracellular stores (Vijayaraghavan et al., 1992; Sharma and Vijayaraghavan, 2003). Calcium entry through nAChRs is also sufficient to activate Ca^2+^-dependent chloride conductances (Mulle et al., 1992; Vernino et al., 1992), which oppose the depolarization caused by nAChR opening. The differential expression of nAChRs coupled with the sequence of excitatory, followed by inhibitory, responses may underlie the ability of nAChRs to differentially modulate neuronal excitability, depending on the target cell, and the strength and timing of the cholinergic input (Frazier et al., 1998; Ji and Dani, 2000; Alkondon and Albuquerque, 2004).

mAChRs are seven transmembrane domain metabotropic receptors and include five pharmacologically defined isoforms—M_1_–M_5_ (Caulfield and Birdsall, 1998). Muscarinic receptors are coupled either to G_q/11_ proteins (M_1_, M_3_, and M_5_) or G_i/o_ proteins (M_2_ and M_4_). Differences in G-protein coupling preferences lie within an amino acid sequence divergence in the third intracellular loop between the M_1_/M_3_/M_5_ sequences compared with the M_2_/M_4_ sequences (Wess et al., 1997). In the hippocampus, M_1_ receptors are widely expressed in somata and dendrites of pyramidal neurons and granule cells, with a small fraction expressed on axons and terminals (Yamasaki et al., 2010). Some studies have also reported expression in interneurons (Cea-Del Rio et al., 2010, 2011). M_2_ receptors are expressed in fibers surrounding pyramidal cells, with the highest density of expression found presynaptically in GABAergic terminals projecting onto the perisomatic region of pyramidal cells (Raiteri et al., 1984; Levey et al., 1995; Hajos et al., 1998; Szabo et al., 2010). There is also immunohistochemical evidence that M_2_ receptors are found postsynaptically in dendrites and cell bodies of interneurons in the stratum oriens and alveus of CA1 (Rouse et al., 1997). M_3_ receptors are expressed at low levels in pyramidal cells and interneurons (Levey et al., 1995; Lawrence et al., 2006; Cea-Del Rio et al., 2010, 2011), whereas M_4_ receptors are enriched in non-pyramidal neurons and in glutamatergic terminals (Levey et al., 1995). M_5_ receptors are only detected at very low levels in the hippocampus (Levey et al., 1995).

In contrast to the fast response produced by activation of nAChRs, mAChR-mediated transmission is slow, owing to their dependence on G-protein-coupled signaling mechanisms (Madison et al., 1987). As a consequence of their pre- and postsynaptic location, muscarinic receptors can have diverse impacts on neuronal activity, influencing the net effect of acetylcholine action. Presynaptic G_i/o_ coupled mAChRs (M_2_, M_4_) cause inhibition of voltage-gated Ca^2+^ channels, a decrease in cAMP-mediated signaling and inhibition of neurotransmitter release at cholinergic, GABAergic and glutamatergic terminals (Zhang et al., 2002; Szabo et al., 2010; Dasari and Gulledge, 2011). Conversely, G_q/11_ coupled postsynaptic mAChRs (M_1_, M_3_, M_5_) potentiate NMDA currents (Markram and Segal, 1990c; Marino et al., 1998; Fernandez De Sevilla et al., 2008), modulate voltage-dependent Ca^2+^ currents (Toselli et al., 1989) and upregulate phopholipase C, inositol trisphosphate and intracellular Ca^2+^ (Power and Sah, 2002; Gulledge and Kawaguchi, 2007). G_q/11_ coupled mAChRs also inhibit potassium conductances including M-current and currents underlying both medium and slow afterhyperpolarization (AHP), causing membrane depolarization and increasing input resistance (Brown and Adams, 1980; Halliwell and Adams, 1982; Cole and Nicoll, 1984; Madison et al., 1987; Buchanan et al., 2010; Giessel and Sabatini, 2010). These receptors are also reported to potentiate the hyperpolarization-activated cation current (I_h_) (Colino and Halliwell, 1993; Fisahn et al., 2002), transient receptor potential (TRP) current (Doerner et al., 2011) and Ca^2+^-dependent non-specific cation current (I_cat_) (Colino and Halliwell, 1993; Fisahn et al., 2002). Interestingly, both mAChRs and nAChRs may also be present on astrocytes leading to intracellular Ca^2+^ rises and neurotransmitter release that can modulate synaptic transmission and plasticity in the hippocampus (Sharma and Vijayaraghavan, 2001; Takata et al., 2011; Shen and Yakel, 2012).

The wide range of receptor subtypes and their localization at both pre- and postsynaptic sites on both excitatory and inhibitory neurons enables the cholinergic system to modulate cellular, synaptic and network activity in the hippocampus. Integrating the functional contribution of fast nicotinic- and slower muscarinic-mediated responses may allow acetylcholine to influence the dynamic properties of hippocampal networks over multiple timescales, creating optimal time windows for the induction of synaptic plasticity and resulting in the emergence of stable oscillatory ensembles, both of which play important roles in hippocampal information processing and memory formation.

***Questions:*** *Why are individual acetylcholine receptor subtypes targeted to specific cellular and subcellular locations? Can different patterns of cholinergic afferent input differentially recruit separate receptor populations and cell types? What role does each receptor subtype perform in hippocampal function?* *What is the overall effect of acetylcholine acting on nicotinic and muscarinic receptors on hippocampal network function?*

## Volume vs. wired transmission

In addition to the anatomical organization of septohippocampal cholinergic projections, it is important to understand the spatiotemporal release and kinetic profiles of acetylcholine within the hippocampus. This has been broadly characterized into wired and volume transmission.

Wired neurotransmission is defined as intercellular communication occurring through a well-defined structure where chemical synapses are adapted to transmit signals through a cascade of events, which result in the activation of ion channels located on the postsynaptic membrane. In this model, the presence and high catalytic activity of acetylcholinesterase (AChE) restricts neurotransmission to classic synapses or junctional complexes. Following axon terminal depolarization, neurotransmitter release and binding to nicotinic and muscarinic acetylcholine receptors, acetylcholine is rapidly hydrolyzed by AChE to yield choline and acetate. Choline is then transported back into the terminal by high-affinity choline transporters to be resynthesized into acetylcholine by the enzyme choline acetyltransferase (ChAT).

Conversely, volume transmission is characterized by multiple extracellular pathways through which signals can diffuse in a three-dimensional fashion and activate extrasynaptic receptors. In this case, presynaptic cholinergic terminals do not make synaptic contacts and therefore do not face a defined postsynaptic density (Vizi and Kiss, 1998; Zoli et al., 1999). As a result, neurotransmission is mediated by acetylcholine that escapes initial hydrolysis by AChE, reaches the extracellular space and stimulates non-junctional nAChRs and mAChRs (Sarter et al., 2009). Importantly, volume and wired transmission are not mutually exclusive.

Septohippocampal cholinergic fibers have been classified into thick, myelinated axons with large terminal boutons present in the hippocampal stratum oriens, stratum radiatum, stratum launosum-moleculare, dentate hilus, and infragranular zone of the dentate gyrus; and thin, unmyelinated varicose fibers found in the hippocampal pyramidal cell layer, dentate granular and molecular layers (Nyakas et al., 1987; Gaykema et al., 1991). The observation that the majority (80–90%) of axon terminals are diffusely organized (Descarries et al., 1997) and do not associate with distinct postsynaptic sites (Houser et al., 1983; Wainer et al., 1984; Frotscher and Leranth, 1985; Vizi and Kiss, 1998) led to the hypothesis that cholinergic transmission in the hippocampus is primarily mediated by volume transmission. Thus, activity of cholinergic projections to the hippocampus was proposed to set a cholinergic “tone,” resulting in an extracellular ambient level of acetylcholine (Descarries, 1998) estimated to be in the low nanomolar range (Vinson and Justice, 1997) although transient concentrations can reach the high nanomolar to low micromolar range (Parikh et al., 2007; Zhang et al., 2010).

Until recently acetylcholine concentrations were measured using microdialysis techniques which have temporal resolutions limited to the minute timescale. As a consequence, the characterization of changes in acetylcholine release associated with brain states were temporally limited, giving rise to the reductionist classification of either “high” or “low” levels of acetylcholine (Marrosu et al., 1995; Yamamuro et al., 1995; Pepeu and Giovannini, 2004; Gold et al., 2011). Improved amperometric techniques now allow for the measurement of acetylcholine concentrations on faster sub-second timescales and have revealed that acetylcholine concentrations in the cortex can fluctuate rapidly with changes in behavioral state (Parikh et al., 2004, 2007; Burmeister et al., 2008; Mattinson et al., 2011; Howe et al., 2013; Paolone et al., 2013). Although acetylcholine concentrations appear to increase during theta oscillations in the hippocampus of anaesthetized rats, it is not clear whether the increase in hippocampal acetylcholine concentration is accompanied by an increase in firing of medial septal cholinergic neurons (Simon et al., 2006; Zhang et al., 2010, 2011) and whether this is tightly correlated with changes in oscillatory activity during the performance of hippocampal-dependent memory tasks.

***Questions:*** *What concentrations of acetylcholine are found in the hippocampus? How do they fluctuate during behavior and on what timescale?* *Are different roles played by volume and wired cholinergic transmission? At which acetylcholine concentrations will distinct types of acetylcholine receptors be activated?*

## Role of the septohippocampal pathway in rhythmic network oscillations

Oscillatory activity is a network phenomenon generated by feedback connections that occur throughout the brain. The connectivity pattern of the hippocampus and medial septum local networks, together with reciprocal connections between the structures, gives rise to the septohippocampal feedback loop. The structural features of this network and the regulation of intrinsic electrophysiological properties of the neuronal populations by acetylcholine are intimately related with the control of hippocampal rhythmic oscillations.

Theta frequency oscillations (4–12 Hz) are particularly prominent in the hippocampus. Two types of hippocampal theta oscillations have been defined—type 1 (7–12 Hz) is associated with voluntary movement and exploratory behavior, whereas type 2 (4–6 Hz) is present during immobility, in particular REM sleep, and occurs spontaneously during urethane anesthesia (Kramis et al., 1975). These two types of hippocampal EEG theta rhythm were distinguished by their relative sensitivities to treatment with atropine, a non-selective competitive antagonist for muscarinic acetylcholine receptors: type 1 was found to be atropine-resistant, whereas type 2 was abolished by atropine (Vanderwolf, 1975; Goutagny et al., 2008). This was consistent with data showing that lesions to septohippocampal neurons caused the loss of bursting activity in the medial septum (Apartis et al., 1998) and theta oscillations in the hippocampus (Rawlins et al., 1979; Colom, 2006). *In vivo* data has also described two types of medial septal rhythmically bursting neurons where, based on their sensitivities to mAChR competitive antagonists atropine or scopolamine, the rhythmicity of one cell type was abolished while the other was unaffected (Brazhnik and Vinogradova, 1986; Stewart and Fox, 1989b,c). The hypothesis that septohippocampal rhythmically bursting neurons were cholinergic and responsible for generating hippocampal theta oscillations was challenged by data showing that both types of theta have atropine-sensitive and resistant components (Brazhnik and Vinogradova, 1986; Stewart and Fox, 1989a). Although selective lesions specifically targeting septohippocampal cholinergic neurons did not completely abolish the two types of theta, the amplitude was significantly reduced, suggesting that cholinergic neurons play a role in regulating, rather than generating, hippocampal theta oscillations (Lee et al., 1994; Bassant et al., 1995; Apartis et al., 1998).

Electrophysiologically, the neuronal cell types populating the medial septum have been classified as slow-firing (~5 Hz) cholinergic, fast- and burst-firing (~10–18 Hz) GABAergic and fast- and cluster-firing (~8–14 Hz) glutamatergic neurons (Griffith and Matthews, 1986; Markram and Segal, 1990b; Jones et al., 1999; Morris et al., 1999; Sotty et al., 2003; Simon et al., 2006; Huh et al., 2010). This classification is consistent with immunohistochemical data and reverse transcription-PCR analysis correlating electrophysiological properties with ChAT, glutamic acid decarboxylase 67 (GAD67) and vesicular glutamate transporters (VGLUT1, VGLUT2) mRNA expression (Sotty et al., 2003). GABAergic neurons display bursting activity at theta frequency which is tightly coupled to hippocampal theta waves (Borhegyi et al., 2004; Bassant et al., 2005; Simon et al., 2006; Hangya et al., 2009). The burst firing of these neurons appears to be dependent on the activation of I_h_, since blocking I_h_ abolishes medial septum interneuron burst firing and hippocampal theta oscillations (Xu et al., 2004). In contrast, the long-duration AHP and slow firing rates characteristic of medial septal cholinergic neurons limit their capacity for theta-related rhythmically bursting activity. Moreover, recordings from cholinergic neurons *in vivo* have so far provided conflicting data with no agreement as to whether cholinergic neurons increase their firing during periods of hippocampal theta activity (Simon et al., 2006; Zhang et al., 2010, 2011). These apparently contradictory features brought forward the question of what is the exact role played by the cholinergic component of the septohippocampal system in the generation of hippocampal theta rhythm. It is now widely accepted that both cholinergic and GABAergic inputs play a role in hippocampal theta oscillations. Medial septal burst-firing GABAergic neurons are key players in generating and maintaining hippocampal theta activity by pacing the activity of GABAergic hippocampal interneurons and, indirectly, of pyramidal cells (Freund and Antal, 1988; Toth et al., 1997; Yoder and Pang, 2005; Goutagny et al., 2008; Hangya et al., 2009). Slow-firing cholinergic cells, in turn, have been proposed to modulate the amplitude of theta (Lee et al., 1994; Apartis et al., 1998).

The mechanisms by which the cholinergic system modulates theta oscillations are not fully understood. M_1_/M_3_ mAChR activation has been shown to increase interneuron firing reliability and sharpen firing precision to theta frequency input, thereby tuning interneurons to amplify theta oscillations (Lawrence et al., 2006). On the other hand, nAChRs have been proposed to modulate pre-existing oscillatory states (Williams and Kauer, 1997; Cobb et al., 1999) by enhancing a slow calcium-dependent potassium conductance that reduces the firing of stratum oriens interneurons (Griguoli et al., 2009). As with any oscillatory network, innervation of GABAergic neurons of the lateral septum by CA1/CA3 principal cells and interneurons (Figure 1) is an essential component of the circuit to transmit rhythmic activity back to the septum (Wang, 2002; Manseau et al., 2008) and to provide a structural basis for feedback regulation of the inhibitory loop. Computational models suggest that this septohippocampal feedback loop relies on the interplay between nAChR- and mAChR- mediated activation and silencing of interneurons to time the occurrence of pyramidal cell activity and to phase theta oscillations during the process of information encoding (Markram et al., 1998; Buzsaki, 2002; Rokers et al., 2002; Wang, 2002; Hasselmo, 2006).

It is important to stress that although isolated hippocampal circuits can generate theta oscillations (Goutagny et al., 2009), hippocampal theta rhythm arises from the coupling of multiple autonomous theta oscillators. Inputs from the entorhinal cortex, activity of the recurrent network of CA3 pyramidal cells and intrinsic resonant properties of hippocampal neurons, all contribute significantly to hippocampal theta oscillations (Buzsaki, 2002; Goutagny et al., 2009). Furthermore, the spontaneous activity of medial septal neurons can be influenced by different inputs from the locus coeruleus, raphe nuclei, and hypothalamus (Segal, 1976; Wilson et al., 1976) suggesting that, in addition to acting as one of several extrinsic rhythm generators that work in concert to amplify and regulate intrinsic theta generators within the hippocampus, the medial septum may serve as a relay and pacemaker station of theta coming from different neighboring areas converging into the medial septum (Dutar et al., 1995). During exploratory behavior, and increased levels of acetylcholine (Marrosu et al., 1995), the synchronization of theta and gamma (30–100 Hz) oscillations has been observed in the hippocampus (Bragin et al., 1995; Csicsvari et al., 2003; Tort et al., 2009). The interaction between these two rhythms has been proposed to create the optimal conditions by which synchrony among neural networks supports synaptic changes necessary for memory formation, storage and retrieval in hippocampal circuits.

In hippocampal slice preparations, gamma oscillations can be induced by application of agonists of muscarinic acetylcholine receptors (Fisahn et al., 1998; Hajos et al., 2004; Mann et al., 2005), kainate receptors (Hajos et al., 2000; Brown et al., 2006), or metabotropic glutamate receptors (Palhalmi et al., 2004). Studies from knock-out mice suggest that the M_1_ receptors mediate cholinergically induced hippocampal gamma (Fisahn et al., 2002). However, it is not clear where these receptors are located (see section on acetylcholine receptors) or whether other mAChR or nAChR subtypes can modulate gamma oscillations.

Although medial septal neuronal populations contribute uniquely to hippocampal rhythmicity, they do so by working in concert with each other. One can therefore propose that the anatomical organization of the septohippocampal system and the interplay between cholinergic, GABAergic and glutamatergic neuronal activities underlie complex, time-dependent excitatory and inhibitory processes where the cholinergic system acts as a modulator of hippocampal theta and gamma oscillatory activity.

***Questions:*** *How do physiological fluctuations in acetylcholine concentration change the electrophysiological properties of hippocampal neurons? How do cholinergic inputs to the hippocampus modulate theta and gamma oscillations? Which acetylcholine receptors present on which cell types are critical for such modulation?*

## Cholinergic regulation of synaptic transmission and plasticity

To appreciate the role of acetylcholine in hippocampal network function we need to understand how acetylcholine regulates synaptic transmission. In the hippocampus, presynaptic acetylcholine receptors modulate neurotransmission in a cell type- and pathway-specific way. *In vitro* studies have demonstrated that acetylcholine can either suppress or enhance excitatory transmission in the hippocampus. Suppression of synaptic transmission at perforant path inputs (Kahle and Cotman, 1989; Foster and Deadwyler, 1992), recurrent CA3 connections (Hasselmo et al., 1995), Schaffer collateral pathway (Qian and Saggau, 1997; Dasari and Gulledge, 2011) and at the connections from CA1 to the subiculum (Kunitake et al., 2004), is achieved by activation of presynaptic mAChRs, most likely M_1_ and M_4_ (Kunitake et al., 2004; Dasari and Gulledge, 2011), that depress presynaptic voltage dependent Ca^2+^ channel activity (Qian and Saggau, 1997) but see (Scanziani et al., 1995). Some evidence is also provided for presynaptic M_2_ receptor-mediated enhancement of voltage-dependent potassium channel activity (Seeger and Alzheimer, 2001). Enhanced transmission results from presynaptic α7 nA ChR activation leading directly to Ca^2+^ influx (Radcliffe et al., 1999). Similarly, presynaptic acetylcholine receptors can also depress or enhance inhibitory transmission. Muscarinic M_2_ receptors inhibit evoked transmission at synapses between parvalbumin positive basket cells and pyramidal cells (Hajos et al., 1998; Szabo et al., 2010). Conversely, nicotinic α3β4 receptors enhance spontaneous transmission at the same synapses (Tang et al., 2011). Presynaptic α4β2 and α7 receptors also enhance transmission at inhibitory synapses (Alkondon et al., 1999; Alkondon and Albuquerque, 2001). Muscarinic M_1_ and/or M_3_ receptors also indirectly inhibit presynaptic release by enhancing synthesis of endocannabinoids and nitric oxide in pyramidal cells that activate CB1 receptors and guanylate cyclase on the terminals of cholecystokinin positive interneurons (Katona et al., 1999; Kim et al., 2002; Ohno-Shosaku et al., 2003; Makara et al., 2007). This is similar, and complementary, to the phenomenon of depolarization-induced suppression of inhibition (Pitler and Alger, 1994; Martin and Alger, 1999; Wilson and Nicoll, 2001; Wilson et al., 2001). As discussed in previous sections, cholinergic activity can also have a dramatic effect on the firing properties of CA1 and CA3 pyramidal cells. This may alter correlated spike activity during rhythmic network oscillations and consequently the induction of synaptic plasticity.

In addition to short-term changes in presynaptic neurotransmitter release, activation of acetylcholine receptors can induce LTP or LTD of synaptic transmission in the hippocampus in a dose dependent manner. Weak mAChR activation leads to LTP and strong mAChR activation to LTD by activation of intracellular signaling pathways (Markram and Segal, 1990c; Auerbach and Segal, 1994, 1996; Scheiderer et al., 2006; Fernandez De Sevilla et al., 2008; Jo et al., 2010).

As well as directly causing short- and long-term synaptic plasticity, cholinergic receptor activation modulates the induction of synaptic plasticity (Shimoshige et al., 1997; Leung et al., 2003; Ovsepian et al., 2004; Ge and Dani, 2005; Shinoe et al., 2005). The precise mechanism and direction of modulation may depend on acetylcholine concentration, the timing of its release, exposure time and the temporal sequence of nAChRs and mAChRs activation in relation to ongoing neuronal activity (Fujii and Sumikawa, 2001; Ge and Dani, 2005; Gu and Yakel, 2011; Gu et al., 2012). A number of mechanisms have been proposed for the modulation of synaptic plasticity. Enhancement of NMDA receptor function via M_1_ receptor-mediated inhibition of small conductance calcium-activated potassium (SK) channels facilitates the induction of LTP (Markram and Segal, 1990a; Buchanan et al., 2010; Giessel and Sabatini, 2010). Modulation of GABAergic inhibition of pyramidal neurons by presynaptic α4β2, α7, or M_2_ receptors (Shimoshige et al., 1997; Ji et al., 2001; Yamazaki et al., 2005) or indirectly via release of endocannabinoids (Carlson et al., 2002; Chevaleyre and Castillo, 2004) modulates the induction of synaptic plasticity at excitatory synapses. Enhancement of postsynaptic excitability by α7 receptors facilitates LTP or LTD depending on the timing of acetylcholine application (Ge and Dani, 2005). Enhancement of action potential generation and backpropagation into dendrites by M_1_ receptor-dependent inhibition of voltage-activated Kv7 potassium channels (Tsubokawa and Ross, 1997; Cho et al., 2008; Petrovic et al., 2012) facilitates LTP. mAChR also enhance dendritic excitability by increasing calcium concentrations in apical dendrites (Power and Sah, 2002; Cho et al., 2008). Finally, M_1_ receptors and NMDA receptors regulate dendritic voltage-gated Kv4.2 potassium channels (Losonczy et al., 2008) that facilitate LTP induction. The existence of multiple mechanisms, each dependent on specific concentrations and timing of acetylcholine release, potentially explains the variety of effects on synaptic plasticity. Many of these mechanisms may also be complementary. For example, the reduction in inhibitory transmission by presynaptic M_2_ receptors coupled with an increase in pyramidal cell excitability by α7 receptors both facilitate the induction of LTP (Ji et al., 2001; Ge and Dani, 2005; Yamazaki et al., 2005). In a further example, it has been shown that LTP can be facilitated and LTD abolished by mAChR activation and that in the presence of higher concentrations of acetylcholine LTD is switched to LTP (Sugisaki et al., 2011).

*In vitro* and *in vivo* studies have supported the idea that theta and gamma oscillations provide a mechanism for bringing together in time glutamatergic inputs to pyramidal cell dendrites and dendritic invasion of fast spikes, the key elements for the induction of synaptic plasticity. Furthermore, inhibition from different classes of interneurons, creating gamma oscillations within each theta cycle and the modulated efficacy of excitatory inputs at different theta phases can selectively influence the timing of pyramidal cell firing (Lengyel et al., 2005). Therefore, promotion of coordinated firing and rhythmic activity by acetylcholine release may provide an increase in the baseline excitability of neurons enhancing responses to glutamate and promoting interactions among neurons that bring about the synaptic changes necessary for memory formation. Within this system, synaptic input that arrives during the positive phase of theta induces LTP whilst input that occurs during the negative phase induces LTD or depotentiation (Greenstein et al., 1988; Huerta and Lisman, 1993, 1995; Holscher et al., 1997; Hyman et al., 2003). In addition, cholinergic receptor activation enhances LTP induction during exploration (Leung et al., 2003) and theta entrained hippocampal place cell activity (Isaac et al., 2009). Therefore, working synergistically with theta, within the optimal time window for STDP, high cholinergic tone during phases of exploration ensures that plasticity is reliably induced.

***Questions:*** *Most studies examining the role of acetylcholine in synaptic transmission and plasticity use exogenous agonist application. Which forms of synaptic transmission and plasticity modulation are engaged by physiological acetylcholine release? What mechanisms are important? Is the physiological timing of cholinergic input important for modulating synaptic plasticity in the hippocampus?*

## New approaches to old problems

Currently, there is considerable focus on dissecting the neuronal substrates of behavior by linking specific cell types and populations to their activity patterns and extending this knowledge to probe the neuronal substrates of behavior. This approach is proving critical for understanding how neuronal circuits contribute to nervous system function.

Although much is known about the effects of acetylcholine in the hippocampus, it is difficult to predict, from the detail of individual receptors and synaptic pathways, what the overall effect will be. To understand this we need better tools to measure cholinergic neuron firing rates and the consequent release of acetylcholine, in addition to specific interventions to disrupt or activate cholinergic input to the hippocampus. The diffuse nature of this cholinergic pathway makes the isolation and experimental stimulation/silencing of well-defined groups of cholinergic fibers difficult to achieve. Recently developed tools could circumvent many of these technical problems and lead to new insights into the role of acetylcholine in modulating network activity, synaptic plasticity and behavioral output.

In this context, bioengineering and genetic tools, combined with transgenic animals, have become a powerful resource for the anatomical and functional deconstruction of neuronal networks. Cell type-specific expression of protein markers and light-gated ion channels allow the structural dynamics and electrical activity of genetically defined neurons to be manipulated and analysed on the millisecond timescale (Luo et al., 2008; Witten et al., 2010; Higley et al., 2011; Yizhar et al., 2011; Kalmbach et al., 2012). Optogenetic approaches are also minimally invasive, add versatility to conventional electrophysiological approaches and circumvent limitations such as difficulties with simultaneous targeting of multiple distinct cell types.

Although optogenetic tools will allow more precise simulation of acetylcholine release, the patterns of release will not be optimal unless there is a better understanding of the physiological patterns of cholinergic cell firing and fluctuations in acetylcholine concentrations in the extracellular space. The ability to mimic *in vivo* patterns of acetylcholine release will be critical for identifying and dissecting the physiological effects of cholinergic neuromodulation in the hippocampus. Two complementary approaches could reveal the temporal specificity of cholinergic neuron firing and subsequent acetylcholine release. The first is accurate measurement of the release kinetics and extracellular concentrations of acetylcholine/choline both *in vivo* and *in vitro* at high temporal resolution. This has been performed in discrete areas of the cortex during different behavioral states but information is not yet available for the hippocampus (Parikh et al., 2004, 2007; Howe et al., 2013; Paolone et al., 2013). Complementary approaches to determine the firing patterns of anatomically and histochemically defined septohippocampal cholinergic neurons will provide additional information on the release of acetylcholine in the hippocampus as a function of behavioral state (Simon et al., 2006). Some data from the hippocampus is already available but the picture is far from complete (Simon et al., 2006; Zhang et al., 2010, 2011).

The combination of these new approaches may provoke a fundamental rethink of the functions of cholinergic inputs to the hippocampus and reveal how specific receptors mediate those functions, therefore presenting an opportunity to establish causal connections between the activity of the septohippocampal cholinergic system, hippocampal network function, learning and memory.

### Conflict of interest statement

The authors declare that the research was conducted in the absence of any commercial or financial relationships that could be construed as a potential conflict of interest.

## References

[B1] Acsady L. Halasy K. Freund T. F. (1993). Calretinin is present in non-pyramidal cells of the rat hippocampus–III. Their inputs from the median raphe and medial septal nuclei. Neuroscience 52, 829–841 10.1016/0306-452290532-K 7680801

[B2] Albuquerque E. X. Pereira E. F. Alkondon M. Rogers S. W. (2009). Mammalian nicotinic acetylcholine receptors: from structure to function. Physiol. Rev. 89, 73–120 10.1152/physrev.00015.2008 19126755 PMC2713585

[B3] Alkondon M. Albuquerque E. X. (2001). Nicotinic acetylcholine receptor alpha7 and alpha4beta2 subtypes differentially control GABAergic input to CA1 neurons in rat hippocampus. J. Neurophysiol. 86, 3043–3055 11731559 10.1152/jn.2001.86.6.3043

[B4] Alkondon M. Albuquerque E. X. (2004). The nicotinic acetylcholine receptor subtypes and their function in the hippocampus and cerebral cortex. Prog. Brain Res. 145, 109–120 10.1016/S0079-612345007-3 14650910

[B5] Alkondon M. Pereira E. F. Eisenberg H. M. Albuquerque E. X. (1999). Choline and selective antagonists identify two subtypes of nicotinic acetylcholine receptors that modulate GABA release from CA1 interneurons in rat hippocampal slices. J. Neurosci. 19, 2693–2705 10087082 10.1523/JNEUROSCI.19-07-02693.1999 PMC6786070

[B6] Alonso A. Kohler C. (1982). Evidence for separate projections of hippocampal pyramidal and non-pyramidal neurons to different parts of the septum in the rat brain. Neurosci. Lett. 31, 209–214 10.1016/0304-394090021-0 7133556

[B7] Amaral D. G. Kurz J. (1985). An analysis of the origins of the cholinergic and noncholinergic septal projections to the hippocampal formation of the rat. J. Comp. Neurol. 240, 37–59 10.1002/cne.902400104 4056104

[B8] Anagnostaras S. G. Murphy G. G. Hamilton S. E. Mitchell S. L. Rahnama N. P. Nathanson N. M. (2003). Selective cognitive dysfunction in acetylcholine M1 muscarinic receptor mutant mice. Nat. Neurosci. 6, 51–58 10.1038/nn992 12483218

[B9] Apartis E. Poindessous-Jazat F. R. Lamour Y. A. Bassant M. H. (1998). Loss of rhythmically bursting neurons in rat medial septum following selective lesion of septohippocampal cholinergic system. J. Neurophysiol. 79, 1633–1642 9535934 10.1152/jn.1998.79.4.1633

[B10] Atri A. Sherman S. Norman K. A. Kirchhoff B. A. Nicolas M. M. Greicius M. D. (2004). Blockade of central cholinergic receptors impairs new learning and increases proactive interference in a word paired-associate memory task. Behav. Neurosci. 118, 223–236 10.1037/0735-7044.118.1.223 14979800

[B11] Auerbach J. M. Segal M. (1994). A novel cholinergic induction of long-term potentiation in rat hippocampus. J. Neurophysiol. 72, 2034–2040 7823117 10.1152/jn.1994.72.4.2034

[B12] Auerbach J. M. Segal M. (1996). Muscarinic receptors mediating depression and long-term potentiation in rat hippocampus. J. Physiol. Lond. 492, 479–493 9019544 10.1113/jphysiol.1996.sp021323 PMC1158842

[B13] Bartus R. T. Dean R. L. 3rd Beer B. Lippa A. S. (1982). The cholinergic hypothesis of geriatric memory dysfunction. Science 217, 408–414 10.1126/science.7046051 7046051

[B14] Bassant M. H. Apartis E. Jazat-Poindessous F. R. Wiley R. G. Lamour Y. A. (1995). Selective immunolesion of the basal forebrain cholinergic neurons: effects on hippocampal activity during sleep and wakefulness in the rat. Neurodegeneration 4, 61–70 10.1006/neur.1995.0007 7600185

[B15] Bassant M. H. Simon A. Poindessous-Jazat F. Csaba Z. Epelbaum J. Dournaud P. (2005). Medial septal GABAergic neurons express the somatostatin sst2A receptor: functional consequences on unit firing and hippocampal theta. J. Neurosci. 25, 2032–2041 10.1523/JNEUROSCI.4619-04.2005 15728843 PMC6726075

[B16] Bell K. A. Shim H. Chen C. K. McQuiston A. R. (2011). Nicotinic excitatory postsynaptic potentials in hippocampal CA1 interneurons are predominantly mediated by nicotinic receptors that contain alpha4 and beta2 subunits. Neuropharmacology 61, 1379–1388 10.1016/j.neuropharm.2011.08.024 21878344 PMC3196356

[B17] Bland B. H. Colom L. V. (1993). Extrinsic and intrinsic properties underlying oscillation and synchrony in limbic cortex. Prog. Neurobiol. 41, 157–208 10.1016/0301-008290007-F 8332751

[B18] Bland B. H. Oddie S. D. Colom L. V. (1999). Mechanisms of neural synchrony in the septohippocampal pathways underlying hippocampal theta generation. J. Neurosci. 19, 3223–3237 10191335 10.1523/JNEUROSCI.19-08-03223.1999 PMC6782275

[B19] Borhegyi Z. Varga V. Szilagyi N. Fabo D. Freund T. F. (2004). Phase segregation of medial septal GABAergic neurons during hippocampal theta activity. J. Neurosci. 24, 8470–8479 10.1523/JNEUROSCI.1413-04.2004 15456820 PMC6729892

[B20] Bragin A. Jando G. Nadasdy Z. Hetke J. Wise K. Buzsaki G. (1995). Gamma (40–100 Hz) oscillation in the hippocampus of the behaving rat. J. Neurosci. 15, 47–60 7823151 10.1523/JNEUROSCI.15-01-00047.1995 PMC6578273

[B21] Brazhnik E. S. Vinogradova O. S. (1986). Control of the neuronal rhythmic bursts in the septal pacemaker of theta-rhythm: effects of anaesthetic and anticholinergic drugs. Brain Res. 380, 94–106 10.1016/0006-899391433-2 3756475

[B22] Brown D. A. Adams P. R. (1980). Muscarinic suppression of a novel voltage-sensitive K+ current in a vertebrate neurone. Nature 283, 673–676 10.1038/283673a0 6965523

[B23] Brown J. T. Teriakidis A. Randall A. D. (2006). A pharmacological investigation of the role of GLUK5-containing receptors in kainate-driven hippocampal gamma band oscillations. Neuropharmacology 50, 47–56 10.1016/j.neuropharm.2005.07.017 16153668

[B24] Buchanan K. A. Petrovic M. M. Chamberlain S. E. Marrion N. V. Mellor J. R. (2010). Facilitation of long-term potentiation by muscarinic M(1) receptors is mediated by inhibition of SK channels. Neuron 68, 948–963 10.1016/j.neuron.2010.11.018 21145007 PMC3003154

[B25] Burmeister J. J. Pomerleau F. Huettl P. Gash C. R. Werner C. E. Bruno J. P. (2008). Ceramic-based multisite microelectrode arrays for simultaneous measures of choline and acetylcholine in CNS. Biosens. Bioelectron. 23, 1382–1389 10.1016/j.bios.2007.12.013 18243683

[B26] Buzsaki G. (2002). Theta oscillations in the hippocampus. Neuron 33, 325–340 10.1016/S0896-627300586-X 11832222

[B27] Buzsaki G. (2005). Theta rhythm of navigation: link between path integration and landmark navigation, episodic and semantic memory. Hippocampus 15, 827–840 10.1002/hipo.20113 16149082

[B28] Buzsaki G. Leung L. W. Vanderwolf C. H. (1983). Cellular bases of hippocampal EEG in the behaving rat. Brain Res. 287, 139–171 6357356 10.1016/0165-0173(83)90037-1

[B29] Carlson G. Wang Y. Alger B. E. (2002). Endocannabinoids facilitate the induction of LTP in the hippocampus. Nat. Neurosci. 5, 723–724 12080342 10.1038/nn879

[B30] Caulfield M. P. Birdsall N. J. (1998). International Union of Pharmacology. XVII. Classification of muscarinic acetylcholine receptors. Pharmacol. Rev. 50, 279–290 9647869

[B31] Cea-Del Rio C. A. Lawrence J. J. Erdelyi F. Szabo G. McBain C. J. (2011). Cholinergic modulation amplifies the intrinsic oscillatory properties of CA1 hippocampal cholecystokinin-positive interneurons. J. Physiol. 589, 609–627 10.1113/jphysiol.2010.199422 21115639 PMC3055546

[B32] Cea-Del Rio C. A. Lawrence J. J. Tricoire L. Erdelyi F. Szabo G. McBain C. J. (2010). M3 muscarinic acetylcholine receptor expression confers differential cholinergic modulation to neurochemically distinct hippocampal basket cell subtypes. J. Neurosci. 30, 6011–6024 10.1523/JNEUROSCI.5040-09.2010 20427660 PMC2883452

[B33] Chevaleyre V. Castillo P. E. (2004). Endocannabinoid-mediated metaplasticity in the hippocampus. Neuron 43, 871–881 10.1016/j.neuron.2004.08.036 15363397

[B34] Cho K. H. Jang H. J. Lee E. H. Yoon S. H. Hahn S. J. Jo Y. H. (2008). Differential cholinergic modulation of Ca2+ transients evoked by backpropagating action potentials in apical and basal dendrites of cortical pyramidal neurons. J. Neurophysiol. 99, 2833–2843 10.1152/jn.00063.2008 18417635

[B35] Cobb S. R. Bulters D. O. Suchak S. Riedel G. Morris R. G. Davies C. H. (1999). Activation of nicotinic acetylcholine receptors patterns network activity in the rodent hippocampus. J. Physiol. 518, 131–140 10.1111/j.1469-7793.1999.0131r.x 10373695 PMC2269408

[B36] Cobb S. R. Davies C. H. (2005). Cholinergic modulation of hippocampal cells and circuits. J. Physiol. Lond. 562, 81–88 10.1113/jphysiol.2004.076539 15528238 PMC1665493

[B37] Cole A. E. Nicoll R. A. (1984). Characterization of a slow cholinergic post-synaptic potential recorded *in vitro* from rat hippocampal pyramidal cells. J. Physiol. Lond. 352, 173–188 6747887 10.1113/jphysiol.1984.sp015285 PMC1193205

[B38] Colino A. Halliwell J. V. (1993). Carbachol potentiates Q current and activates a calcium-dependent non-specific conductance in rat hippocampus *in vitro*. Eur. J. Neurosci. 5, 1198–1209 10.1111/j.1460-9568.1993.tb00974.x 8281323

[B39] Colom L. V. (2006). Septal networks: relevance to theta rhythm, epilepsy and Alzheimer's disease. J. Neurochem. 96, 609–623 10.1111/j.1471-4159.2005.03630.x 16405497

[B40] Csicsvari J. Jamieson B. Wise K. D. Buzsaki G. (2003). Mechanisms of gamma oscillations in the hippocampus of the behaving rat. Neuron 37, 311–322 10.1016/S0896-627301169-8 12546825

[B41] Dani J. A. Bertrand D. (2007). Nicotinic acetylcholine receptors and nicotinic cholinergic mechanisms of the central nervous system. Annu. Rev. Pharmacol. Toxicol. 47, 699–729 10.1146/annurev.pharmtox.47.120505.105214 17009926

[B42] Dasari S. Gulledge A. T. (2011). M1 and M4 receptors modulate hippocampal pyramidal neurons. J. Neurophysiol. 105, 779–792 10.1152/jn.00686.2010 21160001 PMC3059175

[B43] Davis K. L. Mohs R. C. Marin D. Purohit D. P. Perl D. P. Lantz M. (1999). Cholinergic markers in elderly patients with early signs of Alzheimer disease. JAMA 281, 1401–1406 10.1001/jama.281.15.1401 10217056

[B44] De Rosa E. Hasselmo M. E. (2000). Muscarinic cholinergic neuromodulation reduces proactive interference between stored odor memories during associative learning in rats. Behav. Neurosci. 114, 32–41 10.1037/0735-7044.114.1.32 10718260

[B45] Descarries L. (1998). The hypothesis of an ambient level of acetylcholine in the central nervous system. J. Physiol. Paris 92, 215–220 10.1016/S0928-425780013-2 9789811

[B46] Descarries L. Gisiger V. Steriade M. (1997). Diffuse transmission by acetylcholine in the CNS. Prog. Neurobiol. 53, 603–625 10.1016/S0301-008200050-6 9421837

[B47] Doerner J. F. Hatt H. Ramsey I. S. (2011). Voltage- and temperature-dependent activation of TRPV3 channels is potentiated by receptor-mediated PI(4, 5)P2 hydrolysis. J. Gen. Physiol. 137, 271–288 10.1085/jgp.200910388 21321070 PMC3047606

[B48] Drever B. D. Riedel G. Platt B. (2011). The cholinergic system and hippocampal plasticity. Behav. Brain Res. 221, 505–514 10.1016/j.bbr.2010.11.037 21130117

[B49] Dutar P. Bassant M. H. Senut M. C. Lamour Y. (1995). The septohippocampal pathway: structure and function of a central cholinergic system. Physiol. Rev. 75, 393–427 7724668 10.1152/physrev.1995.75.2.393

[B50] Everitt B. J. Robbins T. W. (1997). Central cholinergic systems and cognition. Annu. Rev. Psychol. 48, 649–684 10.1146/annurev.psych.48.1.649 9046571

[B51] Fabian-Fine R. Skehel P. Errington M. L. Davies H. A. Sher E. Stewart M. G. (2001). Ultrastructural distribution of the alpha7 nicotinic acetylcholine receptor subunit in rat hippocampus. J. Neurosci. 21, 7993–8003 11588172 10.1523/JNEUROSCI.21-20-07993.2001 PMC6763871

[B52] Fernandez De Sevilla D. Nunez A. Borde M. Malinow R. Buno W. (2008). Cholinergic-mediated IP3-receptor activation induces long-lasting synaptic enhancement in CA1 pyramidal neurons. J. Neurosci. 28, 1469–1478 10.1523/JNEUROSCI.2723-07.2008 18256268 PMC6671582

[B53] Fibiger H. C. (1991). Cholinergic mechanisms in learning, memory and dementia: a review of recent evidence. Trends Neurosci. 14, 220–223 10.1016/0166-223690117-D 1716012

[B54] Fisahn A. Pike F. G. Buhl E. H. Paulsen O. (1998). Cholinergic induction of network oscillations at 40 Hz in the hippocampus *in vitro*. Nature 394, 186–189 10.1038/28179 9671302

[B55] Fisahn A. Yamada M. Duttaroy A. Gan J. W. Deng C. X. McBain C. J. (2002). Muscarinic induction of hippocampal gamma oscillations requires coupling of the M1 receptor to two mixed cation currents. Neuron 33, 615–624 10.1016/S0896-627300587-1 11856534

[B56] Foster T. C. Deadwyler S. A. (1992). Acetylcholine modulates averaged sensory evoked responses and perforant path evoked field potentials in the rat dentate gyrus. Brain Res. 587, 95–101 10.1016/0006-899391432-E 1525653

[B57] Frazier C. J. Rollins Y. D. Breese C. R. Leonard S. Freedman R. Dunwiddie T. V. (1998). Acetylcholine activates an alpha-bungarotoxin-sensitive nicotinic current in rat hippocampal interneurons, but not pyramidal cells. J. Neurosci. 18, 1187–1195 9454829 10.1523/JNEUROSCI.18-04-01187.1998 PMC6792737

[B58] Freund T. F. Antal M. (1988). GABA-containing neurons in the septum control inhibitory interneurons in the hippocampus. Nature 336, 170–173 10.1038/336170a0 3185735

[B59] Frotscher M. Leranth C. (1985). Cholinergic innervation of the rat hippocampus as revealed by choline acetyltransferase immunocytochemistry: a combined light and electron microscopic study. J. Comp. Neurol. 239, 237–246 10.1002/cne.902390210 4044938

[B60] Fu W. M. Liou H. C. Chen Y. H. (1998). Nerve terminal currents induced by autoreception of acetylcholine release. J. Neurosci. 18, 9954–9961 9822751 10.1523/JNEUROSCI.18-23-09954.1998 PMC6793324

[B61] Fujii S. Sumikawa K. (2001). Nicotine accelerates reversal of long-term potentiation and enhances long-term depression in the rat hippocampal CA1 region. Brain Res. 894, 340–346 10.1016/S0006-899302058-3 11251213

[B62] Gaykema R. P. Van Der Kuil J. Hersh L. B. Luiten P. G. (1991). Patterns of direct projections from the hippocampus to the medial septum-diagonal band complex: anterograde tracing with *Phaseolus vulgaris leucoagglutinin* combined with immunohistochemistry of choline acetyltransferase. Neuroscience 43, 349–360 10.1016/0306-452290299-4 1656317

[B63] Ge S. Dani J. A. (2005). Nicotinic acetylcholine receptors at glutamate synapses facilitate long-term depression or potentiation. J. Neurosci. 25, 6084–6091 10.1523/JNEUROSCI.0542-05.2005 15987938 PMC6725070

[B64] Giessel A. J. Sabatini B. L. (2010). M1 muscarinic receptors boost synaptic potentials and calcium influx in dendritic spines by inhibiting postsynaptic SK channels. Neuron 68, 936–947 10.1016/j.neuron.2010.09.004 21145006 PMC3052967

[B65] Gold P. E. Countryman R. A. Dukala D. Chang Q. (2011). Acetylcholine release in the hippocampus and prelimbic cortex during acquisition of a socially transmitted food preference. Neurobiol. Learn. Mem. 96, 498–503 10.1016/j.nlm.2011.08.004 21907814 PMC3190061

[B66] Goutagny R. Jackson J. Williams S. (2009). Self-generated theta oscillations in the hippocampus. Nat. Neurosci. 12, 1491–1493 10.1038/nn.2440 19881503

[B67] Goutagny R. Manseau F. Jackson J. Danik M. Williams S. (2008). *In vitro* activation of the medial septum-diagonal band complex generates atropine-sensitive and atropine-resistant hippocampal theta rhythm: an investigation using a complete septohippocampal preparation. Hippocampus 18, 531–535 10.1002/hipo.20418 18306282

[B68] Graham A. J. Ray M. A. Perry E. K. Jaros E. Perry R. H. Volsen S. G. (2003). Differential nicotinic acetylcholine receptor subunit expression in the human hippocampus. J. Chem. Neuroanat. 25, 97–113 10.1016/S0891-061800100-X 12663058

[B69] Greenstein Y. J. Pavlides C. Winson J. (1988). Long-term potentiation in the dentate gyrus is preferentially induced at theta rhythm periodicity. Brain Res. 438, 331–334 10.1016/0006-899391358-3 3345440

[B70] Griffith W. H. Matthews R. T. (1986). Electrophysiology of AChE-positive neurons in basal forebrain slices. Neurosci. Lett. 71, 169–174 10.1016/0304-394090553-7 3785743

[B71] Griguoli M. Scuri R. Ragozzino D. Cherubini E. (2009). Activation of nicotinic acetylcholine receptors enhances a slow calcium-dependent potassium conductance and reduces the firing of stratum oriens interneurons. Eur. J. Neurosci. 30, 1011–1022 10.1111/j.1460-9568.2009.06914.x 19735287

[B72] Gritti I. Henny P. Galloni F. Mainville L. Mariotti M. Jones B. E. (2006). Stereological estimates of the basal forebrain cell population in the rat, including neurons containing choline acetyltransferase, glutamic acid decarboxylase or phosphate-activated glutaminase and colocalizing vesicular glutamate transporters. Neuroscience 143, 1051–1064 10.1016/j.neuroscience.2006.09.024 17084984 PMC1831828

[B73] Gu Z. Lamb P. W. Yakel J. L. (2012). Cholinergic coordination of presynaptic and postsynaptic activity induces timing-dependent hippocampal synaptic plasticity. J. Neurosci. 32, 12337–12348 10.1523/JNEUROSCI.2129-12.2012 22956824 PMC3474164

[B74] Gu Z. Yakel J. L. (2011). Timing-dependent septal cholinergic induction of dynamic hippocampal synaptic plasticity. Neuron 71, 155–165 10.1016/j.neuron.2011.04.026 21745645 PMC3134790

[B75] Gulledge A. T. Kawaguchi Y. (2007). Phasic cholinergic signaling in the hippocampus: functional homology with the neocortex. Hippocampus 17, 327–332 10.1002/hipo.20279 17407133

[B76] Gulyas A. I. Gorcs T. J. Freund T. F. (1990). Innervation of different peptide-containing neurons in the hippocampus by GABAergic septal afferents. Neuroscience 37, 31–44 10.1016/0306-452290189-B 1978740

[B77] Gulyas A. I. Hajos N. Katona I. Freund T. F. (2003). Interneurons are the local targets of hippocampal inhibitory cells which project to the medial septum. Eur. J. Neurosci. 17, 1861–1872 10.1046/j.1460-9568.2003.02630.x 12752786

[B78] Hajos N. Katona I. Naiem S. S. Mackie K. Ledent C. Mody I. (2000). Cannabinoids inhibit hippocampal GABAergic transmission and network oscillations. Eur. J. Neurosci. 12, 3239–3249 10.1046/j.1460-9568.2000.00217.x 10998107

[B79] Hajos N. Palhalmi J. Mann E. O. Nemeth B. Paulsen O. Freund T. F. (2004). Spike timing of distinct types of GABAergic interneuron during hippocampal gamma oscillations *in vitro.* J. Neurosci. 24, 9127–9137 10.1523/JNEUROSCI.2113-04.2004 15483131 PMC6730063

[B80] Hajos N. Papp E. C. Acsady L. Levey A. I. Freund T. F. (1998). Distinct interneuron types express m2 muscarinic receptor immunoreactivity on their dendrites or axon terminals in the hippocampus. Neuroscience 82, 355–376 10.1016/S0306-452200300-X 9466448

[B81] Hajszan T. Alreja M. Leranth C. (2004). Intrinsic vesicular glutamate transporter 2-immunoreactive input to septohippocampal parvalbumin-containing neurons: novel glutamatergic local circuit cells. Hippocampus 14, 499–509 10.1002/hipo.10195 15224985

[B82] Halliwell J. V. Adams P. R. (1982). Voltage-clamp analysis of muscarinic excitation in hippocampal neurons. Brain Res. 250, 71–92 10.1016/0006-899390954-4 6128061

[B83] Hangya B. Borhegyi Z. Szilagyi N. Freund T. F. Varga V. (2009). GABAergic neurons of the medial septum lead the hippocampal network during theta activity. J. Neurosci. 29, 8094–8102 10.1523/JNEUROSCI.5665-08.2009 19553449 PMC6666051

[B84] Hasselmo M. E. (2006). The role of acetylcholine in learning and memory. Curr. Opin. Neurobiol. 16, 710–715 10.1016/j.conb.2006.09.002 17011181 PMC2659740

[B85] Hasselmo M. E. Schnell E. Barkai E. (1995). Dynamics of learning and recall at excitatory recurrent synapses and cholinergic modulation in rat hippocampal region CA3. J. Neurosci. 15, 5249–5262 7623149 10.1523/JNEUROSCI.15-07-05249.1995 PMC6577857

[B86] Higley M. J. Gittis A. H. Oldenburg I. A. Balthasar N. Seal R. P. Edwards R. H. (2011). Cholinergic interneurons mediate fast VGluT3-dependent glutamatergic transmission in the striatum. PLoS ONE 6:e19155 10.1371/journal.pone.0019155 21544206 PMC3081336

[B87] Hill J. A. Jr. Zoli M. Bourgeois J. P. Changeux J. P. (1993). Immunocytochemical localization of a neuronal nicotinic receptor: the beta 2-subunit. J. Neurosci. 13, 1551–1568 8463835 10.1523/JNEUROSCI.13-04-01551.1993 PMC6576729

[B88] Holscher C. Anwyl R. Rowan M. J. (1997). Stimulation on the positive phase of hippocampal theta rhythm induces long-term potentiation that can be depotentiated by stimulation on the negative phase in area CA1 *in vivo*. J. Neurosci. 17, 6470–6477 9236254 10.1523/JNEUROSCI.17-16-06470.1997 PMC6568346

[B89] Houser C. R. Crawford G. D. Barber R. P. Salvaterra P. M. Vaughn J. E. (1983). Organization and morphological characteristics of cholinergic neurons: an immunocytochemical study with a monoclonal antibody to choline acetyltransferase. Brain Res. 266, 97–119 10.1016/0006-899391312-4 6850348

[B90] Howe W. M. Berry A. S. Francois J. Gilmour G. Carp J. M. Tricklebank M. (2013). Prefrontal cholinergic mechanisms instigating shifts from monitoring for cues to cue-guided performance: converging electrochemical and FMRI evidence from rats and humans. J. Neurosci. 33, 8742–8752 10.1523/JNEUROSCI.5809-12.2013 23678117 PMC3690786

[B91] Huerta P. T. Lisman J. E. (1993). heightened synaptic plasticity of hippocampal ca1 neurons during a cholinergically induced rhythmic state. Nature 364, 723–725 10.1038/364723a0 8355787

[B92] Huerta P. T. Lisman J. E. (1995). Bidirectional synaptic plasticity induced by a single burst during cholinergic theta-oscillation in ca1 *in-vitro*. Neuron 15, 1053–1063 10.1016/0896-627390094-2 7576649

[B93] Huh C. Y. Goutagny R. Williams S. (2010). Glutamatergic neurons of the mouse medial septum and diagonal band of Broca synaptically drive hippocampal pyramidal cells: relevance for hippocampal theta rhythm. J. Neurosci. 30, 15951–15961 10.1523/JNEUROSCI.3663-10.2010 21106833 PMC6633737

[B94] Hyman J. M. Wyble B. P. Goyal V. Rossi C. A. Hasselmo M. E. (2003). Stimulation in hippocampal region CA1 in behaving rats yields long-term potentiation when delivered to the peak of theta and long-term depression when delivered to the trough. J. Neurosci. 23, 11725–11731 14684874 10.1523/JNEUROSCI.23-37-11725.2003 PMC6740943

[B95] Isaac J. T. Buchanan K. A. Muller R. U. Mellor J. R. (2009). Hippocampal place cell firing patterns can induce long-term synaptic plasticity *in vitro.* J. Neurosci. 29, 6840–6850 10.1523/JNEUROSCI.0731-09.2009 19474311 PMC2692101

[B96] Ji D. Dani J. A. (2000). Inhibition and disinhibition of pyramidal neurons by activation of nicotinic receptors on hippocampal interneurons. J. Neurophysiol. 83, 2682–2690 10805668 10.1152/jn.2000.83.5.2682

[B97] Ji D. Lape R. Dani J. A. (2001). Timing and location of nicotinic activity enhances or depresses hippocampal synaptic plasticity. Neuron 31, 131–141 10.1016/S0896-627300332-4 11498056

[B98] Jo J. Son G. H. Winters B. L. Kim M. J. Whitcomb D. J. Dickinson B. A. (2010). Muscarinic receptors induce LTD of NMDAR EPSCs via a mechanism involving hippocalcin, AP2 and PSD-95. Nat. Neurosci. 13, 1216–1224 10.1038/nn.2636 20852624

[B99] Jones G. A. Norris S. K. Henderson Z. (1999). Conduction velocities and membrane properties of different classes of rat septohippocampal neurons recorded *in vitro.* J. Physiol. 517, 867–877 10.1111/j.1469-7793.1999.0867s.x 10358125 PMC2269364

[B100] Kahle J. S. Cotman C. W. (1989). Carbachol depresses synaptic responses in the medial but not the lateral perforant path. Brain Res. 482, 159–163 10.1016/0006-899390554-4 2706473

[B101] Kalmbach A. Hedrick T. Waters J. (2012). Selective optogenetic stimulation of cholinergic axons in neocortex. J. Neurophysiol. 107, 2008–2019 10.1152/jn.00870.2011 22236708 PMC3331667

[B102] Katona I. Sperlagh B. Sik A. Kafalvi A. Vizi E. S. Mackie K. (1999). Presynaptically located CB1 cannabinoid receptors regulate GABA release from axon terminals of specific hippocampal interneurons. J. Neurosci. 19, 4544–4558 10341254 10.1523/JNEUROSCI.19-11-04544.1999 PMC6782612

[B103] Katz P. S. (2011). Neural mechanisms underlying the evolvability of behaviour. Philos. Trans. R. Soc. Lond. B Biol. Sci. 366, 2086–2099 10.1098/rstb.2010.0336 21690127 PMC3130364

[B104] Kawai H. Zago W. Berg D. K. (2002). Nicotinic alpha 7 receptor clusters on hippocampal GABAergic neurons: regulation by synaptic activity and neurotrophins. J. Neurosci. 22, 7903–7912 12223543 10.1523/JNEUROSCI.22-18-07903.2002 PMC6758091

[B105] Kelsey J. E. Landry B. A. (1988). Medial septal lesions disrupt spatial mapping ability in rats. Behav. Neurosci. 102, 289–293 10.1037/0735-7044.102.2.289 3365323

[B106] Kim J. Isokawa M. Ledent C. Alger B. E. (2002). Activation of muscarinic acetylcholine receptors enhances the release of endogenous cannabinoids in the hippocampus. J. Neurosci. 22, 10182–10191 12451119 10.1523/JNEUROSCI.22-23-10182.2002 PMC6758770

[B107] Kramis R. Vanderwolf C. H. Bland B. H. (1975). Two types of hippocampal rhythmical slow activity in both the rabbit and the rat: relations to behavior and effects of atropine, diethyl ether, urethane, and pentobarbital. Exp. Neurol. 49, 58–85 10.1016/0014-488690195-8 1183532

[B108] Kunitake A. Kunitake T. Stewart M. (2004). Differential modulation by carbachol of four separate excitatory afferent systems to the rat subiculum *in vitro*. Hippocampus 14, 986–999 10.1002/hipo.20016 15390173

[B109] Lawrence J. J. Grinspan Z. M. Statland J. M. McBain C. J. (2006). Muscarinic receptor activation tunes mouse stratum oriens interneurones to amplify spike reliability. J. Physiol. 571, 555–562 10.1113/jphysiol.2005.103218 16439425 PMC1805794

[B110] Lecourtier L. De Vasconcelos A. P. Leroux E. Cosquer B. Geiger K. Lithfous S. (2011). Septohippocampal pathways contribute to system consolidation of a spatial memory: sequential implication of GABAergic and cholinergic neurons. Hippocampus 21, 1277–1289 10.1002/hipo.20837 20623740

[B111] Lee M. G. Chrobak J. J. Sik A. Wiley R. G. Buzsaki G. (1994). Hippocampal theta activity following selective lesion of the septal cholinergic system. Neuroscience 62, 1033–1047 10.1016/0306-452290341-7 7845584

[B112] Lena C. Changeux J. P. Mulle C. (1993). Evidence for “preterminal” nicotinic receptors on GABAergic axons in the rat interpeduncular nucleus. J. Neurosci. 13, 2680–2688 8501532 10.1523/JNEUROSCI.13-06-02680.1993 PMC6576498

[B113] Lengyel M. Kwag J. Paulsen O. Dayan P. (2005). Matching storage and recall: hippocampal spike timing-dependent plasticity and phase response curves. Nat. Neurosci. 8, 1677–1683 10.1038/nn1561 16261136

[B114] Leranth C. Deller T. Buzsaki G. (1992). Intraseptal connections redefined: lack of a lateral septum to medial septum path. Brain Res. 583, 1–11 10.1016/S0006-899380004-6 1380395

[B115] Leranth C. Frotscher M. (1987). Cholinergic innervation of hippocampal GAD- and somatostatin-immunoreactive commissural neurons. J. Comp. Neurol. 261, 33–47 10.1002/cne.902610104 2887594

[B116] Leranth C. Frotscher M. (1989). Organization of the septal region in the rat brain: cholinergic-GABAergic interconnections and the termination of hippocampo-septal fibers. J. Comp. Neurol. 289, 304–314 10.1002/cne.902890210 2808769

[B117] Leung L. S. Shen B. X. Rajakumar N. Ma J. Y. (2003). Cholinergic activity enhances hippocampal long-term potentiation in CA1 during walking in rats. J. Neurosci. 23, 9297–9304 14561856 10.1523/JNEUROSCI.23-28-09297.2003 PMC6740561

[B118] Levey A. I. Edmunds S. M. Koliatsos V. Wiley R. G. Heilman C. J. (1995). Expression of m1-m4 muscarinic acetylcholine receptor proteins in rat hippocampus and regulation by cholinergic innervation. J. Neurosci. 15, 4077–4092 7751967 10.1523/JNEUROSCI.15-05-04077.1995 PMC6578239

[B118a] Levey A. I. Kitt C. A. Simonds W. F. Price D. L. Brann M. R. (1991). Identification and localization of muscarinic acetylcholine receptor proteins in brain with subtype-specific antibodies. J. Neurosci. 11, 3218–3226 1941081 10.1523/JNEUROSCI.11-10-03218.1991 PMC6575445

[B119] Lewis P. R. Shute C. C. (1967). The cholinergic limbic system: projections to hippocampal formation, medial cortex, nuclei of the ascending cholinergic reticular system, and the subfornical organ and supra-optic crest. Brain 90, 521–540 10.1093/brain/90.3.521 6058141

[B120] Losonczy A. Makara J. K. Magee J. C. (2008). Compartmentalized dendritic plasticity and input feature storage in neurons. Nature 452, 436–440 10.1038/nature06725 18368112

[B121] Luo L. Callaway E. M. Svoboda K. (2008). Genetic dissection of neural circuits. Neuron 57, 634–660 10.1016/j.neuron.2008.01.002 18341986 PMC2628815

[B122] Madison D. V. Lancaster B. Nicoll R. A. (1987). Voltage clamp analysis of cholinergic action in the hippocampus. J. Neurosci. 7, 733–741 3559710 10.1523/JNEUROSCI.07-03-00733.1987 PMC6569053

[B123] Makara J. K. Katona I. Nyiri G. Nemeth B. Ledent C. Watanabe M. (2007). Involvement of nitric oxide in depolarization-induced suppression of inhibition in hippocampal pyramidal cells during activation of cholinergic receptors. J. Neurosci. 27, 10211–10222 10.1523/JNEUROSCI.2104-07.2007 17881527 PMC6672656

[B124] Mann E. O. Suckling J. M. Hajos N. Greenfield S. A. Paulsen O. (2005). Perisomatic feedback inhibition underlies cholinergically induced fast network oscillations in the rat hippocampus *in vitro*. Neuron 45, 105–117 10.1016/j.neuron.2004.12.016 15629706

[B125] Manns I. D. Mainville L. Jones B. E. (2001). Evidence for glutamate, in addition to acetylcholine and GABA, neurotransmitter synthesis in basal forebrain neurons projecting to the entorhinal cortex. Neuroscience 107, 249–263 10.1016/S0306-452200302-5 11731099

[B126] Manseau F. Danik M. Williams S. (2005). A functional glutamatergic neurone network in the medial septum and diagonal band area. J. Physiol. 566, 865–884 10.1113/jphysiol.2005.089664 15919710 PMC1464770

[B127] Manseau F. Goutagny R. Danik M. Williams S. (2008). The hippocamposeptal pathway generates rhythmic firing of GABAergic neurons in the medial septum and diagonal bands: an investigation using a complete septohippocampal preparation *in vitro.* J. Neurosci. 28, 4096–4107 10.1523/JNEUROSCI.0247-08.2008 18400909 PMC6670476

[B128] Marder E. (2012). Neuromodulation of neuronal circuits: back to the future. Neuron 76, 1–11 10.1016/j.neuron.2012.09.010 23040802 PMC3482119

[B129] Margiotta J. F. Berg D. K. Dionne V. E. (1987). Cyclic AMP regulates the proportion of functional acetylcholine receptors on chicken ciliary ganglion neurons. Proc. Natl. Acad. Sci. U.S.A. 84, 8155–8159 10.1073/pnas.84.22.8155 2446319 PMC299497

[B130] Marino M. J. Rouse S. T. Levey A. I. Potter L. T. Conn P. J. (1998). Activation of the genetically defined m1 muscarinic receptor potentiates N-methyl-D-aspartate (NMDA) receptor currents in hippocampal pyramidal cells. Proc. Natl. Acad. Sci. U.S.A. 95, 11465–11470 10.1073/pnas.95.19.11465 9736760 PMC21666

[B131] Markram H. Gupta A. Uziel A. Wang Y. Tsodyks M. (1998). Information processing with frequency-dependent synaptic connections. Neurobiol. Learn. Mem. 70, 101–112 10.1006/nlme.1998.3841 9753590

[B132] Markram H. Segal M. (1990a). Acetylcholine potentiates responses to N-methyl-D-aspartate in the rat hippocampus. Neurosci. Lett. 113, 62–65 10.1016/0304-394090495-U 1973273

[B133] Markram H. Segal M. (1990b). Electrophysiological characteristics of cholinergic and non-cholinergic neurons in the rat medial septum-diagonal band complex. Brain Res. 513, 171–174 10.1016/0006-899391106-Q 2350680

[B134] Markram H. Segal M. (1990c). Long-lasting facilitation of excitatory postsynaptic potentials in the rat hippocampus by acetylcholine. J. Physiol. 427, 381–393 2145426 10.1113/jphysiol.1990.sp018177 PMC1189936

[B135] Marrosu F. Portas C. Mascia M. S. Casu M. A. Fa M. Giagheddu M. (1995). Microdialysis measurement of cortical and hippocampal acetylcholine release during sleep-wake cycle in freely moving cats. Brain Res. 671, 329–332 10.1016/0006-899301399-3 7743225

[B136] Martin L. A. Alger B. E. (1999). Muscarinic facilitation of the occurrence of depolarization-induced suppression of inhibition in rat hippocampus. Neuroscience 92, 61–71 10.1016/S0306-452200745-3 10392830

[B137] Matthews D. A. Salvaterra P. M. Crawford G. D. Houser C. R. Vaughn J. E. (1987). An immunocytochemical study of choline acetyltransferase-containing neurons and axon terminals in normal and partially deafferented hippocampal formation. Brain Res. 402, 30–43 10.1016/0006-899391044-4 3548884

[B138] Mattinson C. E. Burmeister J. J. Quintero J. E. Pomerleau F. Huettl P. Gerhardt G. A. (2011). Tonic and phasic release of glutamate and acetylcholine neurotransmission in sub-regions of the rat prefrontal cortex using enzyme-based microelectrode arrays. J. Neurosci. Methods 202, 199–208 10.1016/j.jneumeth.2011.08.020 21896284 PMC3607211

[B139] McGaughy J. Everitt B. J. Robbins T. W. Sarter M. (2000). The role of cortical cholinergic afferent projections in cognition: impact of new selective immunotoxins. Behav. Brain Res. 115, 251–263 10.1016/S0166-432800262-X 11000424

[B140] McGehee D. S. Heath M. J. Gelber S. Devay P. Role L. W. (1995). Nicotine enhancement of fast excitatory synaptic transmission in CNS by presynaptic receptors. Science 269, 1692–1696 10.1126/science.7569895 7569895

[B141] McKinney M. Coyle J. T. Hedreen J. C. (1983). Topographic analysis of the innervation of the rat neocortex and hippocampus by the basal forebrain cholinergic system. J. Comp. Neurol. 217, 103–121 10.1002/cne.902170109 6875049

[B142] McMahan R. W. Sobel T. J. Baxter M. G. (1997). Selective immunolesions of hippocampal cholinergic input fail to impair spatial working memory. Hippocampus 7, 130–136 9136045 10.1002/(SICI)1098-1063(1997)7:2<130::AID-HIPO2>3.0.CO;2-R

[B143] McQuiston A. R. Madison D. V. (1999). Nicotinic receptor activation excites distinct subtypes of interneurons in the rat hippocampus. J. Neurosci. 19, 2887–2896 10191306 10.1523/JNEUROSCI.19-08-02887.1999 PMC6782295

[B144] Meibach R. C. Siegel A. (1977). Efferent connections of the septal area in the rat: an analysis utilizing retrograde and anterograde transport methods. Brain Res. 119, 1–20 10.1016/0006-899390088-9 63306

[B145] Micheau J. Marighetto A. (2011). Acetylcholine and memory: a long, complex and chaotic but still living relationship. Behav. Brain Res. 221, 424–429 10.1016/j.bbr.2010.11.052 21130809

[B146] Milner T. A. Loy R. Amaral D. G. (1983). An anatomical study of the development of the septo-hippocampal projection in the rat. Brain Res. 284, 343–371 6871729 10.1016/0165-3806(83)90017-2

[B147] Morris N. P. Harris S. J. Henderson Z. (1999). Parvalbumin-immunoreactive, fast-spiking neurons in the medial septum/diagonal band complex of the rat: intracellular recordings *in vitro*. Neuroscience 92, 589–600 10.1016/S0306-452200026-3 10408608

[B148] Muir J. L. (1997). Acetylcholine, aging, and Alzheimer's disease. Pharmacol. Biochem. Behav. 56, 687–696 10.1016/S0091-305700431-5 9130295

[B149] Mulle C. Choquet D. Korn H. Changeux J. P. (1992). Calcium influx through nicotinic receptor in rat central neurons: its relevance to cellular regulation. Neuron 8, 135–143 10.1016/0896-627390115-T 1309647

[B150] Nyakas C. Luiten P. G. Spencer D. G. Traber J. (1987). Detailed projection patterns of septal and diagonal band efferents to the hippocampus in the rat with emphasis on innervation of CA1 and dentate gyrus. Brain Res. Bull. 18, 533–545 10.1016/0361-923090117-1 3607523

[B151] Ohno-Shosaku T. Matsui M. Fukudome Y. Shosaku J. Tsubokawa H. Taketo M. M. (2003). Postsynaptic M1 and M3 receptors are responsible for the muscarinic enhancement of retrograde endocannabinoid signalling in the hippocampus. Eur. J. Neurosci. 18, 109–116 10.1046/j.1460-9568.2003.02732.x 12859343

[B152] Ovsepian S. V. Anwyl R. Rowan M. J. (2004). Endogenous acetylcholine lowers the threshold for long-term potentiation induction in the CA1 area through muscarinic receptor activation: *in vivo* study. Eur. J. Neurosci. 20, 1267–1275 10.1111/j.1460-9568.2004.03582.x 15341598

[B153] Palhalmi J. Paulsen O. Freund T. F. Hajos N. (2004). Distinct properties of carbachol- and DHPG-induced network oscillations in hippocampal slices. Neuropharmacology 47, 381–389 10.1016/j.neuropharm.2004.04.010 15275827

[B154] Paolone G. Angelakos C. C. Meyer P. J. Robinson T. E. Sarter M. (2013). cholinergic control over attention in rats prone to attribute incentive salience to reward cues. J. Neurosci. 33, 8321–8335 10.1523/JNEUROSCI.0709-13.2013 23658172 PMC3690461

[B155] Papp E. C. Hajos N. Acsady L. Freund T. F. (1999). Medial septal and median raphe innervation of vasoactive intestinal polypeptide-containing interneurons in the hippocampus. Neuroscience 90, 369–382 10.1016/S0306-452200455-2 10215142

[B156] Parikh V. Kozak R. Martinez V. Sarter M. (2007). Prefrontal acetylcholine release controls cue detection on multiple timescales. Neuron 56, 141–154 10.1016/j.neuron.2007.08.025 17920021 PMC2084212

[B157] Parikh V. Pomerleau F. Huettl P. Gerhardt G. A. Sarter M. Bruno J. P. (2004). Rapid assessment of *in vivo* cholinergic transmission by amperometric detection of changes in extracellular choline levels. Eur. J. Neurosci. 20, 1545–1554 10.1111/j.1460-9568.2004.03614.x 15355321

[B158] Pepeu G. Blandina P. (1998). The acetylcholine, GABA, glutamate triangle in the rat forebrain. J. Physiol. Paris 92, 351–355 10.1016/S0928-425780004-7 9789836

[B159] Pepeu G. Giovannini M. G. (2004). Changes in acetylcholine extracellular levels during cognitive processes. Learn. Mem. 11, 21–27 10.1101/lm.68104 14747513

[B160] Petrovic M. M. Nowacki J. Olivo V. Tsaneva-Atanaseva K. Randall A. D. Mellor J. R. (2012). Inhibition of post-synaptic Kv7/KCNQ/M channels facilitates long-term potentiation in the hippocampus. PLoS ONE 7:e30402 10.1371/journal.pone.0030402 22348007 PMC3278412

[B161] Pitler T. A. Alger B. E. (1994). Depolarization-induced suppression of GABAergic inhibition in rat hippocampal pyramidal cells: G protein involvement in a presynaptic mechanism. Neuron 13, 1447–1455 10.1016/0896-627390430-8 7993636

[B162] Power J. M. Sah P. (2002). Nuclear calcium signaling evoked by cholinergic stimulation in hippocampal CA1 pyramidal neurons. J. Neurosci. 22, 3454–3462 11978822 10.1523/JNEUROSCI.22-09-03454.2002 PMC6758367

[B163] Qian J. Saggau P. (1997). Presynaptic inhibition of synaptic transmission in the rat hippocampus by activation of muscarinic receptors: involvement of presynaptic calcium influx. Br. J. Pharmacol. 122, 511–519 10.1038/sj.bjp.0701400 9351508 PMC1564959

[B164] Radcliffe K. A. Fisher J. L. Gray R. Dani J. A. (1999). Nicotinic modulation of glutamate and GABA synaptic transmission of hippocampal neurons. Ann. N.Y. Acad. Sci. 868, 591–610 10.1111/j.1749-6632.1999.tb11332.x 10414340

[B165] Raedler T. J. Bymaster F. P. Tandon R. Copolov D. Dean B. (2007). Towards a muscarinic hypothesis of schizophrenia. Mol. Psychiatry 12, 232–246 17146471 10.1038/sj.mp.4001924

[B166] Raiteri M. Leardi R. Marchi M. (1984). Heterogeneity of presynaptic muscarinic receptors regulating neurotransmitter release in the rat brain. J. Pharmacol. Exp. Ther. 228, 209–214 6141277

[B167] Rawlins J. N. Feldon J. Gray J. A. (1979). Septo-hippocampal connections and the hippocampal theta rhythm. Exp. Brain Res. 37, 49–63 10.1007/BF01474253 385334

[B167a] Reever C. M. Ferrari-DiLeo G. Flynn D. D. (1997). The M5 (m5) receptor subtype: fact or fiction? Life Sci. 60, 1105–1112 10.1016/S0024-3205(97)00054-4 9121354

[B168] Risold P. Y. Swanson L. W. (1997). Connections of the rat lateral septal complex. Brain Res. Brain Res. Rev. 24, 115–195 10.1016/S0165-017300009-X 9385454

[B169] Rokers B. Mercado E. 3rd. Allen M. T. Myers C. E. Gluck M. A. (2002). A connectionist model of septohippocampal dynamics during conditioning: closing the loop. Behav. Neurosci. 116, 48–62 10.1037/0735-7044.116.1.48 11895183

[B170] Rouse S. T. Thomas T. M. Levey A. I. (1997). Muscarinic acetylcholine receptor subtype, m2: diverse functional implications of differential synaptic localization. Life Sci. 60, 1031–1038 10.1016/S0024-320500044-1 9121344

[B171] Sakanaka M. Shiosaka S. Takagi H. Senba E. Takatsuki K. Inagaki S. (1980). Topographic organization of the projection from the forebrain subcortical areas to the hippocampal formation of the rat. Neurosci. Lett. 20, 253–257 10.1016/0304-394090156-1 7443075

[B172] Sargent P. B. (1993). The diversity of neuronal nicotinic acetylcholine receptors. Annu. Rev. Neurosci. 16, 403–443 10.1146/annurev.ne.16.030193.002155 7681637

[B173] Sarter M. Parikh V. Howe W. M. (2009). Phasic acetylcholine release and the volume transmission hypothesis: time to move on. Nat. Rev. Neurosci. 10, 383–390 10.1038/nrn2635 19377503 PMC2699581

[B174] Scanziani M. Gahwiler B. H. Thompson S. M. (1995). Presynaptic inhibition of excitatory synaptic transmission by muscarinic and metabotropic glutamate receptor activation in the hippocampus: are Ca2+ channels involved. Neuropharmacology 34, 1549–1557 10.1016/0028-390800119-Q 8606802

[B175] Scheiderer C. L. McCutchen E. Thacker E. E. Kolasa K. Ward M. K. Parsons D. (2006). Sympathetic sprouting drives hippocampal cholinergic reinnervation that prevents loss of a muscarinic receptor-dependent long-term depression at CA3-CA1 synapses. J. Neurosci. 26, 3745–3756 10.1523/JNEUROSCI.5507-05.2006 16597728 PMC6674126

[B176] Schliebs R. Arendt T. (2011). The cholinergic system in aging and neuronal degeneration. Behav. Brain Res. 221, 555–563 10.1016/j.bbr.2010.11.058 21145918

[B177] Schwerdtfeger W. K. Buhl E. (1986). Various types of non-pyramidal hippocampal neurons project to the septum and contralateral hippocampus. Brain Res. 386, 146–154 10.1016/0006-899390151-4 3779406

[B178] Seeger T. Alzheimer C. (2001). Muscarinic activation of inwardly rectifying K(+) conductance reduces EPSPs in rat hippocampal CA1 pyramidal cells. J. Physiol. 535, 383–396 10.1111/j.1469-7793.2001.00383.x 11533131 PMC2278799

[B179] Segal M. (1976). Brain stem afferents to the rat medial septum. J. Physiol. 261, 617–631 185368 10.1113/jphysiol.1976.sp011577 PMC1309163

[B180] Sharma G. Vijayaraghavan S. (2001). Nicotinic cholinergic signaling in hippocampal astrocytes involves calcium-induced calcium release from intracellular stores. Proc. Natl. Acad. Sci. U.S.A. 98, 4148–4153 10.1073/pnas.071540198 11259680 PMC31194

[B181] Sharma G. Vijayaraghavan S. (2003). Modulation of presynaptic store calcium induces release of glutamate and postsynaptic firing. Neuron 38, 929–939 10.1016/S0896-627300322-2 12818178

[B182] Shen J. X. Yakel J. L. (2012). Functional alpha7 nicotinic ACh receptors on astrocytes in rat hippocampal CA1 slices. J. Mol. Neurosci. 48, 14–21 10.1007/s12031-012-9719-3 22351110 PMC3530828

[B183] Shimoshige Y. Maeda T. Kaneko S. Akaike A. Satoh M. (1997). Involvement of M2 receptor in an enhancement of long-term potentiation by carbachol in Schaffer collateral-CA1 synapses of hippocampal slices. Neurosci. Res. 27, 175–180 10.1016/S0168-010201147-9 9100260

[B184] Shinoe T. Matsui M. Taketo M. M. Manabe T. (2005). Modulation of synaptic plasticity by physiological activation of M-1 muscarinic acetylcholine receptors in the mouse hippocampus. J. Neurosci. 25, 11194–11200 10.1523/JNEUROSCI.2338-05.2005 16319319 PMC6725656

[B185] Simon A. P. Poindessous-Jazat F. Dutar P. Epelbaum J. Bassant M. H. (2006). Firing properties of anatomically identified neurons in the medial septum of anesthetized and unanesthetized restrained rats. J. Neurosci. 26, 9038–9046 10.1523/JNEUROSCI.1401-06.2006 16943562 PMC6675331

[B186] Sotty F. Danik M. Manseau F. Laplante F. Quirion R. Williams S. (2003). Distinct electrophysiological properties of glutamatergic, cholinergic and GABAergic rat septohippocampal neurons: novel implications for hippocampal rhythmicity. J. Physiol. 551, 927–943 10.1113/jphysiol.2003.046847 12865506 PMC2343277

[B187] Stewart M. Fox S. E. (1989a). Detection of an atropine-resistant component of the hippocampal theta rhythm in urethane-anesthetized rats. Brain Res. 500, 55–60 10.1016/0006-899390299-0 2605509

[B188] Stewart M. Fox S. E. (1989b). Firing relations of medial septal neurons to the hippocampal theta rhythm in urethane anesthetized rats. Exp. Brain Res. 77, 507–516 10.1007/BF00249604 2806444

[B189] Stewart M. Fox S. E. (1989c). Two populations of rhythmically bursting neurons in rat medial septum are revealed by atropine. J. Neurophysiol. 61, 982–993 2723736 10.1152/jn.1989.61.5.982

[B190] Stewart M. Fox S. E. (1990). Do septal neurons pace the hippocampal theta rhythm. Trends Neurosci. 13, 163–168 10.1016/0166-223690040-H 1693232

[B191] Sugisaki E. Fukushima Y. Tsukada M. Aihara T. (2011). Cholinergic modulation on spike timing-dependent plasticity in hippocampal CA1 network. Neuroscience 192, 91–101 10.1016/j.neuroscience.2011.06.064 21736924

[B192] Szabo G. G. Holderith N. Gulyas A. I. Freund T. F. Hajos N. (2010). Distinct synaptic properties of perisomatic inhibitory cell types and their different modulation by cholinergic receptor activation in the CA3 region of the mouse hippocampus. Eur. J. Neurosci. 31, 2234–2246 10.1111/j.1460-9568.2010.07292.x 20529124 PMC2916217

[B193] Takacs V. T. Freund T. F. Gulyas A. I. (2008). Types and synaptic connections of hippocampal inhibitory neurons reciprocally connected with the medial septum. Eur. J. Neurosci. 28, 148–164 10.1111/j.1460-9568.2008.06319.x 18662340

[B194] Takata N. Mishima T. Hisatsune C. Nagai T. Ebisui E. Mikoshiba K. (2011). Astrocyte calcium signaling transforms cholinergic modulation to cortical plasticity *in vivo.* J. Neurosci. 31, 18155–18165 10.1523/JNEUROSCI.5289-11.2011 22159127 PMC6634158

[B195] Tang A. H. Karson M. A. Nagode D. A. McIntosh J. M. Uebele V. N. Renger J. J. (2011). Nerve terminal nicotinic acetylcholine receptors initiate quantal GABA release from perisomatic interneurons by activating axonal T-type (Cav3) Ca(2)(+) channels and Ca(2)(+) release from stores. J. Neurosci. 31, 13546–13561 10.1523/JNEUROSCI.2781-11.2011 21940446 PMC3353409

[B196] Thompson C. L. Pathak S. D. Jeromin A. Ng L. L. Macpherson C. R. Mortrud M. T. (2008). Genomic anatomy of the hippocampus. Neuron 60, 1010–1021 10.1016/j.neuron.2008.12.008 19109908

[B197] Tort A. B. Komorowski R. W. Manns J. R. Kopell N. J. Eichenbaum H. (2009). Theta-gamma coupling increases during the learning of item-context associations. Proc. Natl. Acad. Sci. U.S.A. 106, 20942–20947 10.1073/pnas.0911331106 19934062 PMC2791641

[B198] Toselli M. Lang J. Costa T. Lux H. D. (1989). Direct modulation of voltage-dependent calcium channels by muscarinic activation of a pertussis toxin-sensitive G-protein in hippocampal neurons. Pflugers Arch. 415, 255–261 10.1007/BF00370874 2560167

[B200] Toth K. Freund T. F. (1992). Calbindin D28k-containing nonpyramidal cells in the rat hippocampus: their immunoreactivity for GABA and projection to the medial septum. Neuroscience 49, 793–805 10.1016/0306-452290357-8 1279455

[B199] Toth K. Borhegyi Z. Freund T. F. (1993). Postsynaptic targets of GABAergic hippocampal neurons in the medial septum-diagonal band of broca complex. J. Neurosci. 13, 3712–3724 7690065 10.1523/JNEUROSCI.13-09-03712.1993 PMC6576440

[B201] Toth K. Freund T. F. Miles R. (1997). Disinhibition of rat hippocampal pyramidal cells by GABAergic afferents from the septum. J. Physiol. 500(Pt 2), 463–474 9147330 10.1113/jphysiol.1997.sp022033 PMC1159396

[B202] Tsubokawa H. Ross W. N. (1997). Muscarinic modulation of spike backpropagation in the apical dendrites of hippocampal CA1 pyramidal neurons. J. Neurosci. 17, 5782–5791 9221776 10.1523/JNEUROSCI.17-15-05782.1997 PMC6573202

[B203] Vanderwolf C. H. (1975). Neocortical and hippocampal activation relation to behavior: effects of atropine, eserine, phenothiazines, and amphetamine. J. Comp. Physiol. Psychol. 88, 300–323 10.1037/h0076211 235571

[B204] Vernino S. Amador M. Luetje C. W. Patrick J. Dani J. A. (1992). Calcium modulation and high calcium permeability of neuronal nicotinic acetylcholine receptors. Neuron 8, 127–134 10.1016/0896-627390114-S 1370370

[B205] Vijayaraghavan S. Pugh P. C. Zhang Z. W. Rathouz M. M. Berg D. K. (1992). Nicotinic receptors that bind alpha-bungarotoxin on neurons raise intracellular free Ca2+. Neuron 8, 353–362 10.1016/0896-627390301-S 1310863

[B206] Vinson P. N. Justice J. B. Jr. (1997). Effect of neostigmine on concentration and extraction fraction of acetylcholine using quantitative microdialysis. J. Neurosci. Methods 73, 61–67 10.1016/S0165-027002213-3 9130679

[B207] Vizi E. S. Kiss J. P. (1998). Neurochemistry and pharmacology of the major hippocampal transmitter systems: synaptic and nonsynaptic interactions. Hippocampus 8, 566–607 9882017 10.1002/(SICI)1098-1063(1998)8:6<566::AID-HIPO2>3.0.CO;2-W

[B208] Wainer B. H. Bolam J. P. Freund T. F. Henderson Z. Totterdell S. Smith A. D. (1984). Cholinergic synapses in the rat brain: a correlated light and electron microscopic immunohistochemical study employing a monoclonal antibody against choline acetyltransferase. Brain Res. 308, 69–76 10.1016/0006-899390918-1 6478204

[B208a] Wall S. J. Wolfe B. B. Kromer L. F. (1994). Cholinergic deafferentation of dorsal hippocampus by fimbria-fornix lesioning differentially regulates subtypes (m1–m5) of muscarinic receptors. J. Neurochem. 62, 1345–1351 10.1046/j.1471-4159.1994.62041345.x 8133265

[B209] Wanaverbecq N. Semyanov A. Pavlov I. Walker M. C. Kullmann D. M. (2007). Cholinergic axons modulate GABAergic signaling among hippocampal interneurons via postsynaptic alpha 7 nicotinic receptors. J. Neurosci. 27, 5683–5693 10.1523/JNEUROSCI.1732-07.2007 17522313 PMC2889598

[B210] Wang X. J. (2002). Pacemaker neurons for the theta rhythm and their synchronization in the septohippocampal reciprocal loop. J. Neurophysiol. 87, 889–900 11826054 10.1152/jn.00135.2001

[B211] Warburton E. C. Koder T. Cho K. Massey P. V. Duguid G. Barker G. R. (2003). Cholinergic neurotransmission is essential for perirhinal cortical plasticity and recognition memory. Neuron 38, 987–996 10.1016/S0896-627300358-1 12818183

[B212] Wess J. (2004). Muscarinic acetylcholine receptor knockout mice: novel phenotypes and clinical implications. Annu. Rev. Pharmacol. Toxicol. 44, 423–450 10.1146/annurev.pharmtox.44.101802.121622 14744253

[B213] Wess J. (2012). Novel muscarinic receptor mutant mouse models. Handb. Exp. Pharmacol. 208, 95–117 10.1007/978-3-642-23274-9_6 22222697

[B214] Wess J. Liu J. Blin N. Yun J. Lerche C. Kostenis E. (1997). Structural basis of receptor/G protein coupling selectivity studied with muscarinic receptors as model systems. Life Sci. 60, 1007–1014 10.1016/S0024-320500041-6 9121341

[B215] Williams J. H. Kauer J. A. (1997). Properties of carbachol-induced oscillatory activity in rat hippocampus. J. Neurophysiol. 78, 2631–2640 9356412 10.1152/jn.1997.78.5.2631

[B216] Wilson C. L. Motter B. C. Lindsley D. B. (1976). Influences of hypothalamic stimulation upon septal and hippocampal electrical activity in the cat. Brain Res. 107, 55–68 10.1016/0006-899390095-0 1268724

[B217] Wilson R. I. Kunos G. Nicoll R. A. (2001). Presynaptic specificity of endocannabinoid signaling in the hippocampus. Neuron 31, 453–462 10.1016/S0896-627300372-5 11516401

[B218] Wilson R. I. Nicoll R. A. (2001). Endogenous cannabinoids mediate retrograde signalling at hippocampal synapses. Nature 410, 588–592 10.1038/35069076 11279497

[B219] Witten I. B. Lin S. C. Brodsky M. Prakash R. Diester I. Anikeeva P. (2010). Cholinergic interneurons control local circuit activity and cocaine conditioning. Science 330, 1677–1681 10.1126/science.1193771 21164015 PMC3142356

[B220] Wonnacott S. (1997). Presynaptic nicotinic ACh receptors. Trends Neurosci. 20, 92–98 10.1016/S0166-223610073-4 9023878

[B221] Xu C. Datta S. Wu M. Alreja M. (2004). Hippocampal theta rhythm is reduced by suppression of the H-current in septohippocampal GABAergic neurons. Eur. J. Neurosci. 19, 2299–2309 10.1111/j.0953-816X.2004.03316.x 15090056

[B222] Yamamuro Y. Hori K. Tanaka J. Iwano H. Nomura M. (1995). Septo-hippocampal cholinergic system under the discrimination learning task in the rat: a microdialysis study with the dual-probe approach. Brain Res. 684, 1–7 10.1016/0006-899300290-7 7583196

[B223] Yamano M. Luiten P. G. (1989). Direct synaptic contacts of medial septal efferents with somatostatin immunoreactive neurons in the rat hippocampus. Brain Res. Bull. 22, 993–1001 10.1016/0361-923090011-7 2571399

[B224] Yamasaki M. Matsui M. Watanabe M. (2010). Preferential localization of muscarinic M1 receptor on dendritic shaft and spine of cortical pyramidal cells and its anatomical evidence for volume transmission. J. Neurosci. 30, 4408–4418 10.1523/JNEUROSCI.5719-09.2010 20335477 PMC6634497

[B225] Yamazaki Y. Jia Y. Hamaue N. Sumikawa K. (2005). Nicotine-induced switch in the nicotinic cholinergic mechanisms of facilitation of long-term potentiation induction. Eur. J. Neurosci. 22, 845–860 10.1111/j.1460-9568.2005.04259.x 16115208

[B226] Yizhar O. Fenno L. E. Davidson T. J. Mogri M. Deisseroth K. (2011). Optogenetics in neural systems. Neuron 71, 9–34 10.1016/j.neuron.2011.06.004 21745635

[B227] Yoder R. M. Pang K. C. (2005). Involvement of GABAergic and cholinergic medial septal neurons in hippocampal theta rhythm. Hippocampus 15, 381–392 10.1002/hipo.20062 15630696

[B228] Zhang H. Lin S. C. Nicolelis M. A. (2010). Spatiotemporal coupling between hippocampal acetylcholine release and theta oscillations *in vivo*. J. Neurosci. 30, 13431–13440 10.1523/JNEUROSCI.1144-10.2010 20926669 PMC2988451

[B229] Zhang H. Lin S. C. Nicolelis M. A. (2011). A distinctive subpopulation of medial septal slow-firing neurons promote hippocampal activation and theta oscillations. J. Neurophysiol. 106, 2749–2763 10.1152/jn.00267.2011 21865435 PMC3214118

[B230] Zhang W. Basile A. S. Gomeza J. Volpicelli L. A. Levey A. I. Wess J. (2002). Characterization of central inhibitory muscarinic autoreceptors by the use of muscarinic acetylcholine receptor knock-out mice. J. Neurosci. 22, 1709–1717 11880500 10.1523/JNEUROSCI.22-05-01709.2002 PMC6758851

[B231] Zoli M. Jansson A. Sykova E. Agnati L. F. Fuxe K. (1999). Volume transmission in the CNS and its relevance for neuropsychopharmacology. Trends Pharmacol. Sci. 20, 142–150 10.1016/S0165-614701343-7 10322499

